# Supplementary material for: A 3K Axiom SNP array from a transcriptome-wide SNP resource sheds new light on the genetic diversity and structure of the iconic subtropical conifer tree Araucaria angustifolia (Bert.) Kuntze
Source: PLoS One. 2020 Aug 31;15(8):e0230404. doi: 10.1371/journal.pone.0230404 (PMC7458329; doi:10.1371/journal.pone.0230404)
Supplement: S6 File — (PDF) [file pone.0230404.s006.pdf]

CLUMPAK main pipeline - Job 1581444186 summary

Major modes for the uploaded data:

K=1

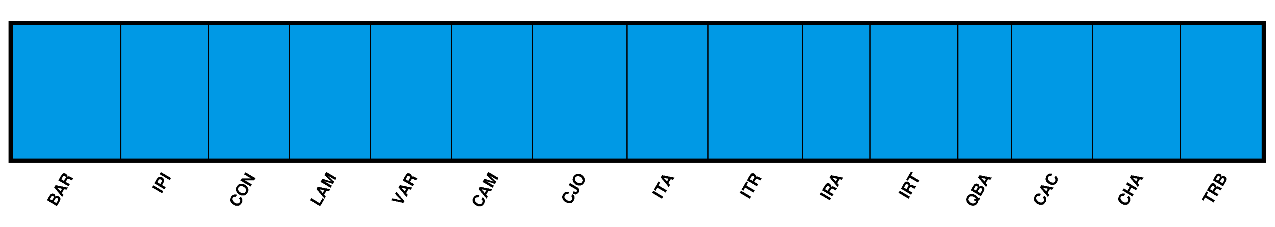

K=2

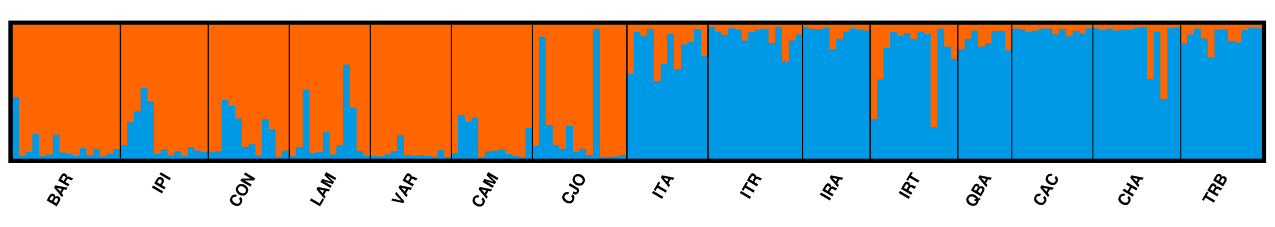

K=3

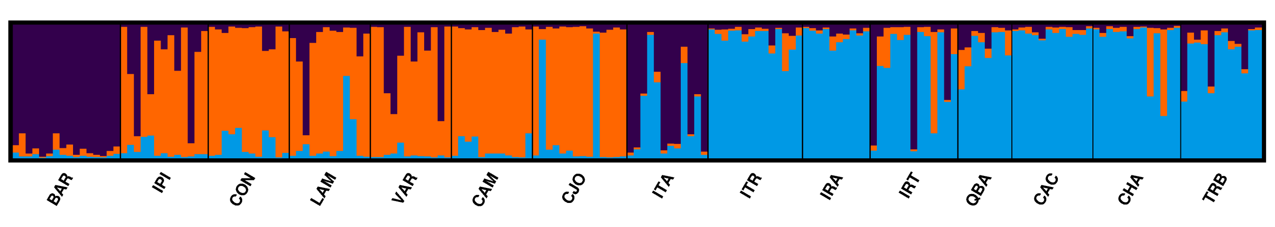

K=4

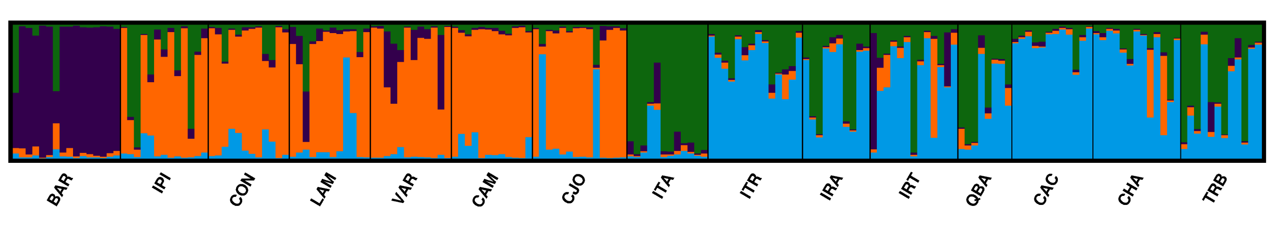

K=5

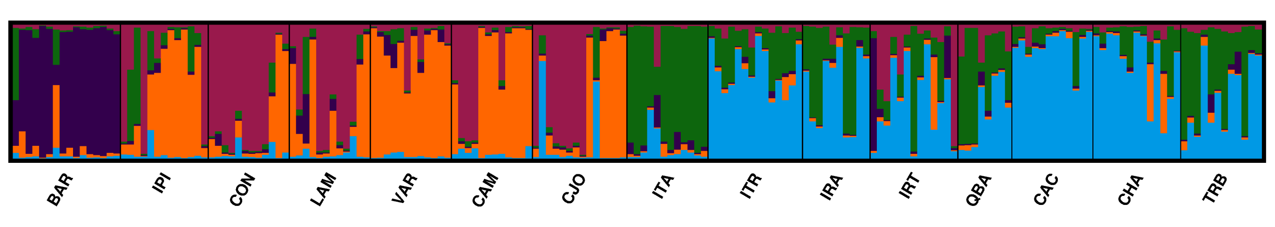

K=6

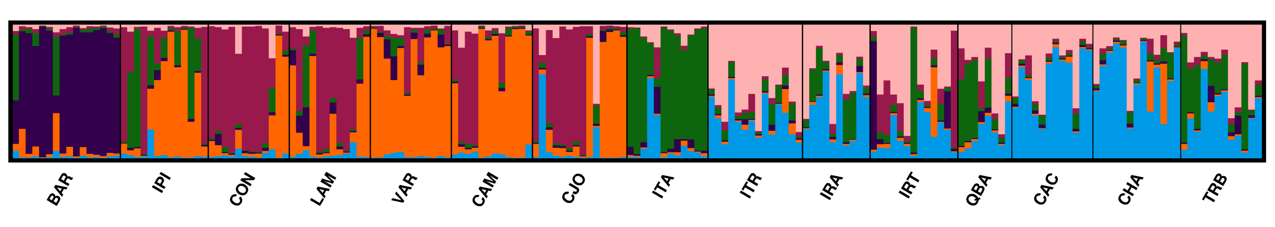

K=7

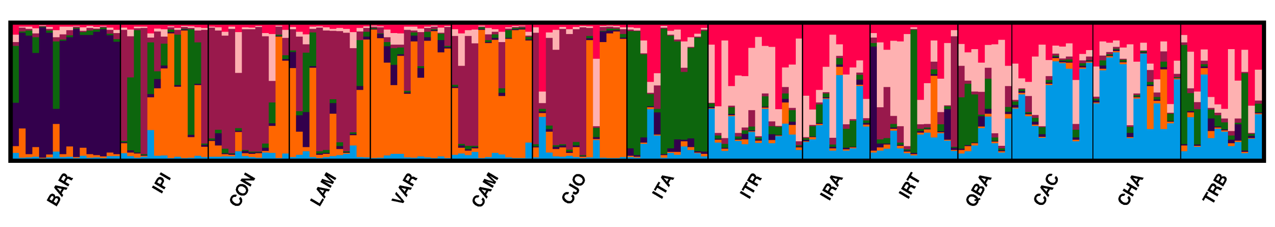

K=8

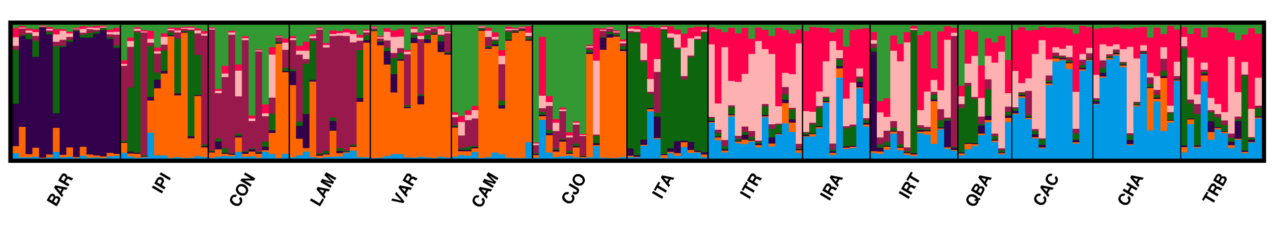

K=9

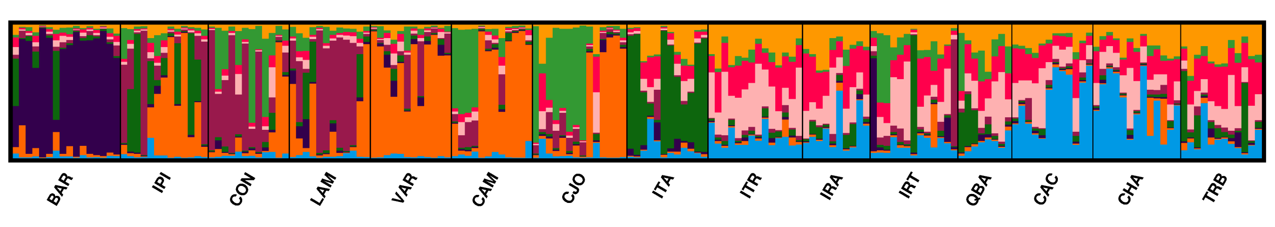

K=10

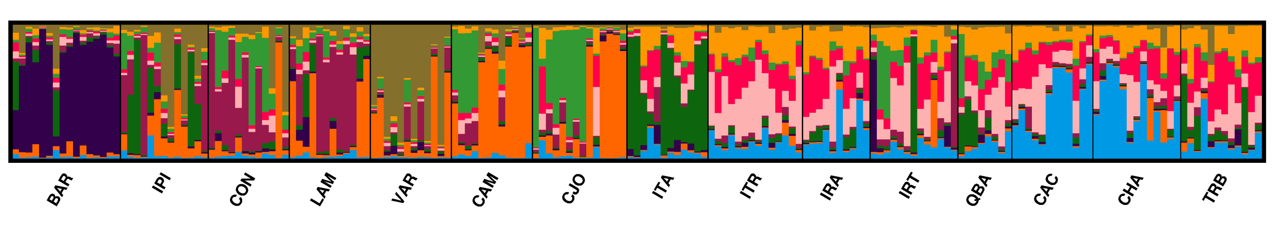

K=11

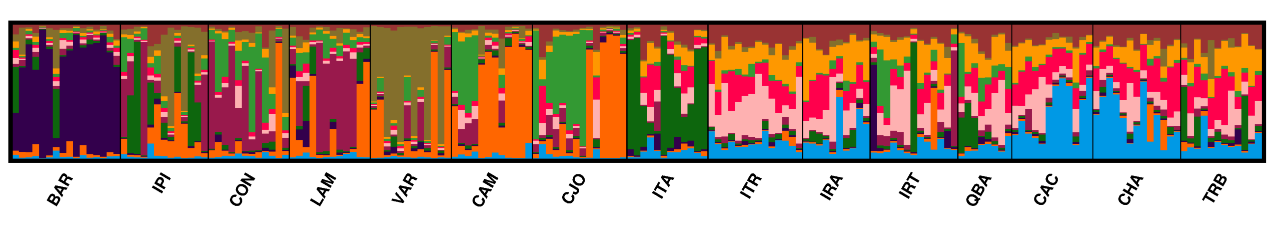

K=12

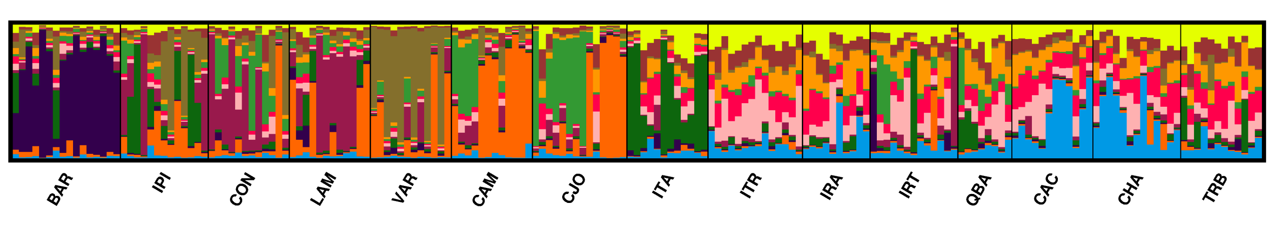

K=13

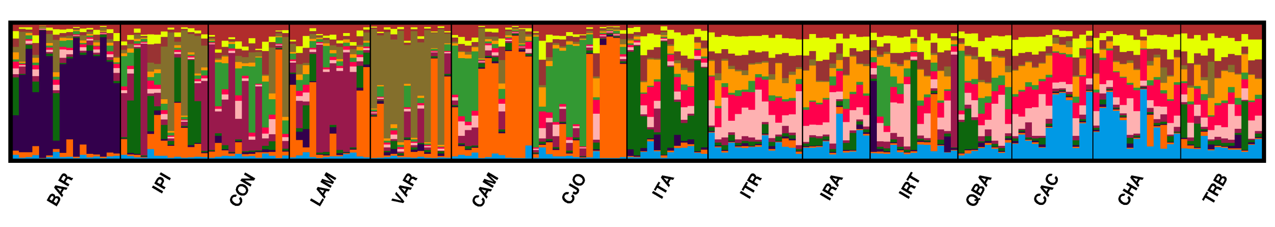

K=14

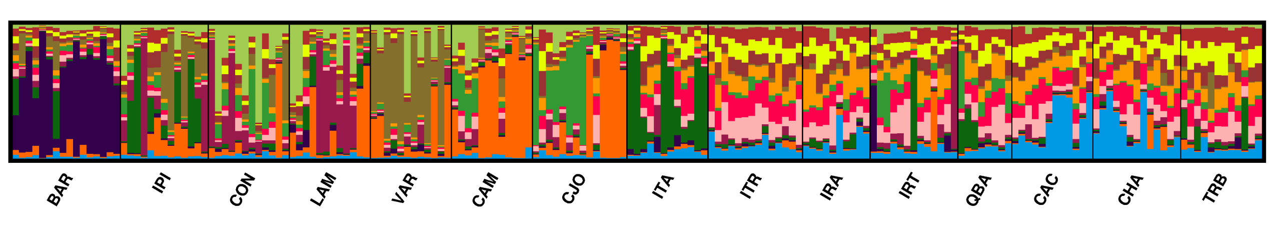

K=15

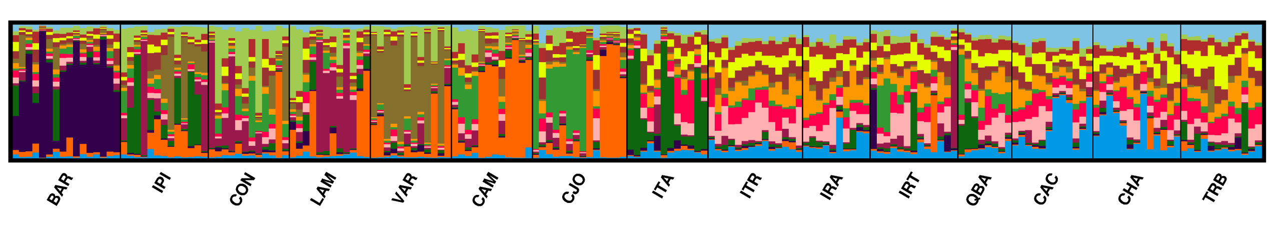

Minor modes for the uploaded data:

K=4 MinorCluster1

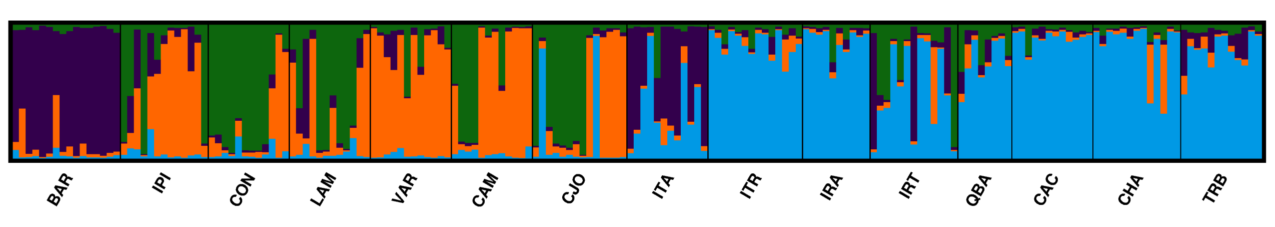

K=7 MinorCluster1

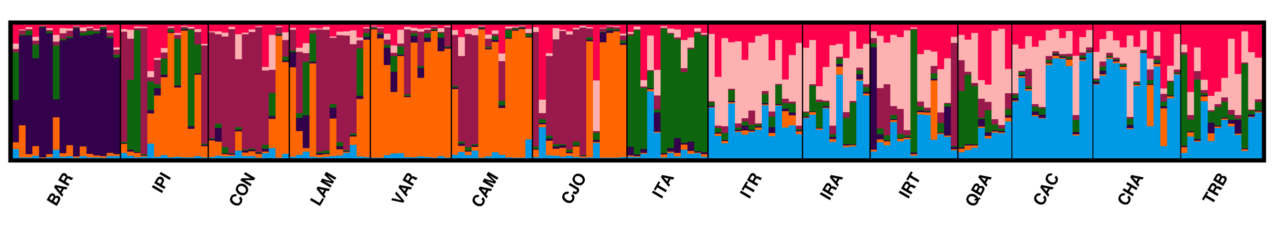

K=8 MinorCluster1

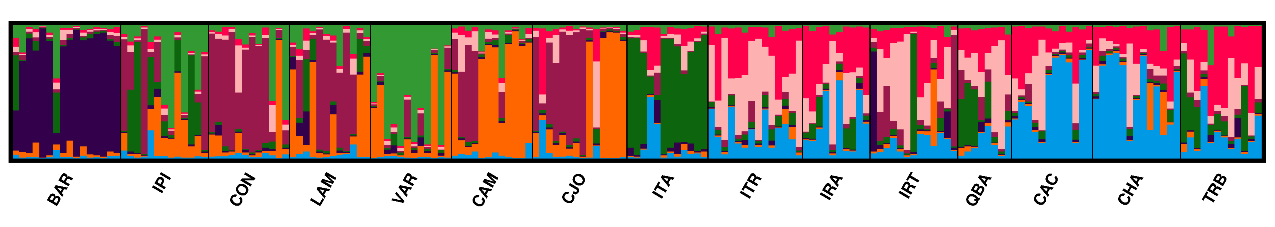

K=8 MinorCluster2

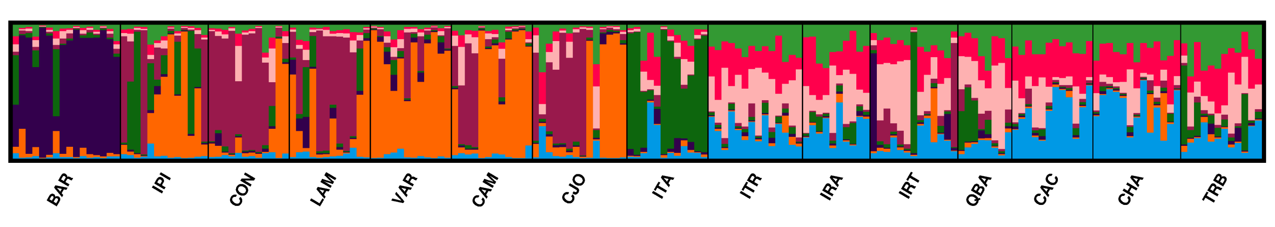

K=9 MinorCluster1

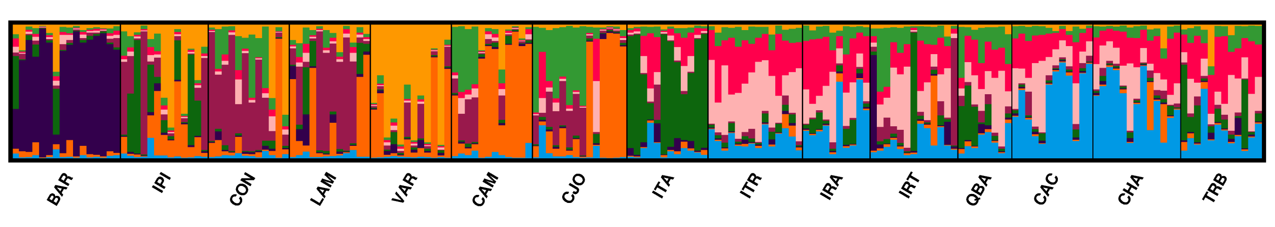

K=10 MinorCluster1

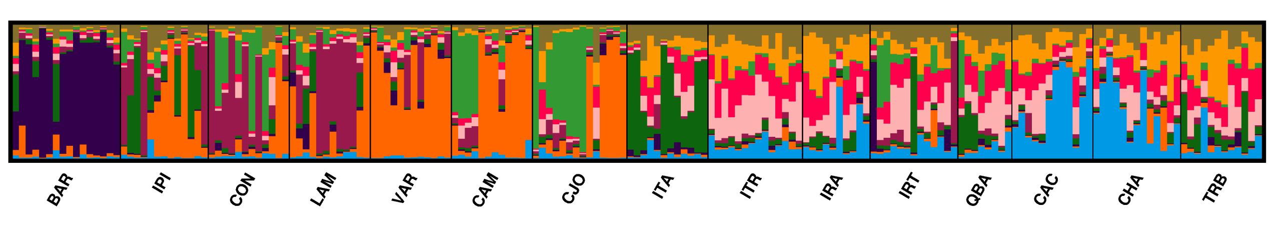

K=12 MinorCluster1

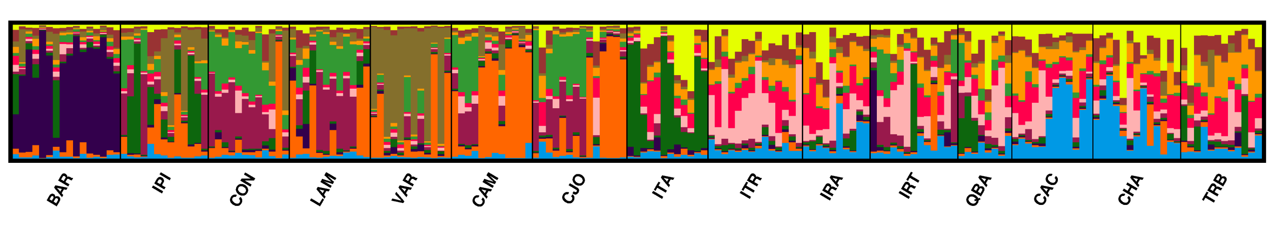

K=13 MinorCluster1

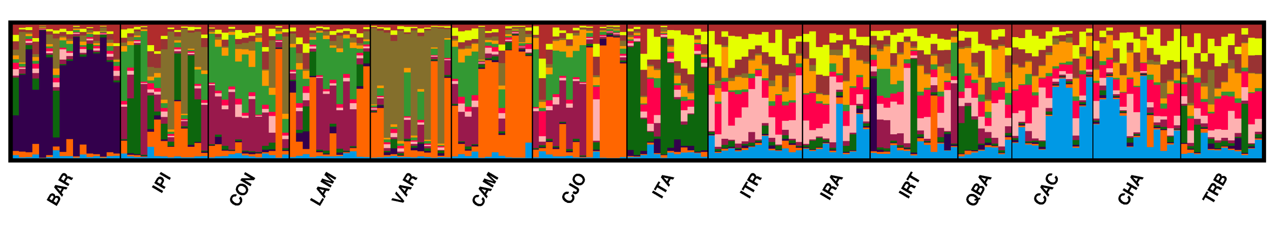

K=14 MinorCluster1

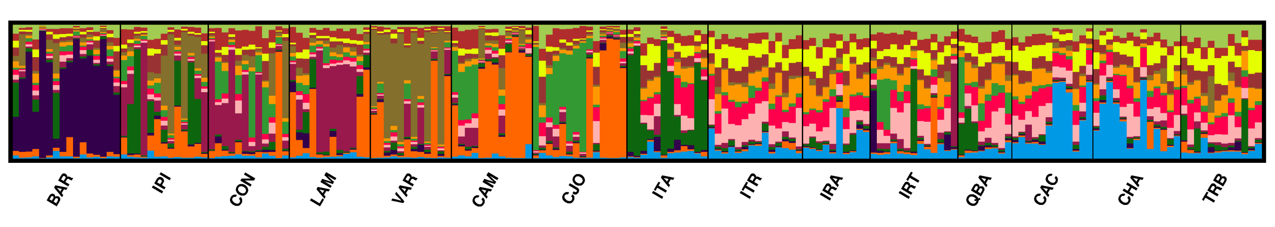

K=15 MinorCluster1

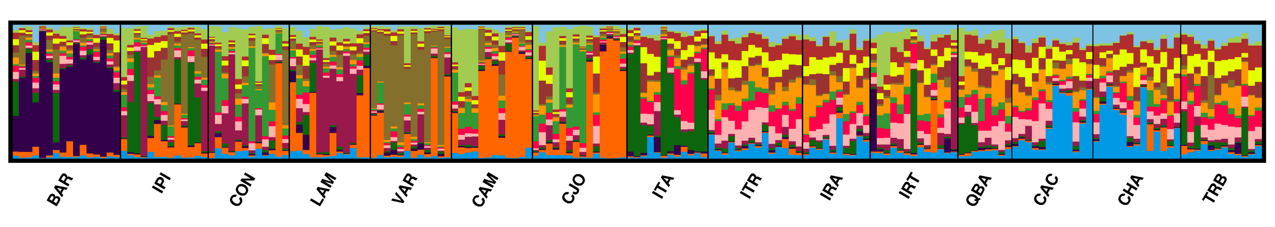

Division of runs by mode:

K=1 21/21
K=2 20/21
K=3 20/20
K=4 13/20, 7/20
K=5 20/20
K=6 19/20
K=7 18/20, 2/20
K=8 12/20, 5/20, 3/20
K=9 11/20, 9/20
K=10 17/20, 3/20
K=11 19/20
K=12 16/20, 2/20
K=13 17/20, 3/20
K=14 12/20, 8/20
K=15 17/20, 3/20
