# Supplementary material for: A 3K Axiom SNP array from a transcriptome-wide SNP resource sheds new light on the genetic diversity and structure of the iconic subtropical conifer tree Araucaria angustifolia (Bert.) Kuntze
Source: PLoS One. 2020 Aug 31;15(8):e0230404. doi: 10.1371/journal.pone.0230404 (PMC7458329; doi:10.1371/journal.pone.0230404)
Supplement: S7 File — (PDF) [file pone.0230404.s007.pdf]

# CLUMPAK main pipeline - Job 1581596881 summary

Major modes for the uploaded data:

K=1

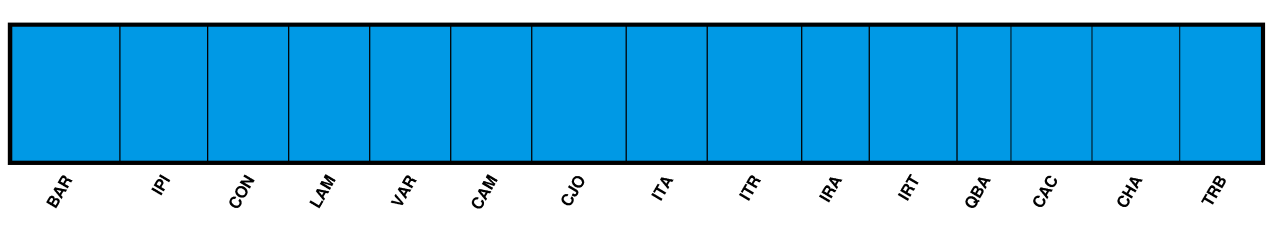


K=2

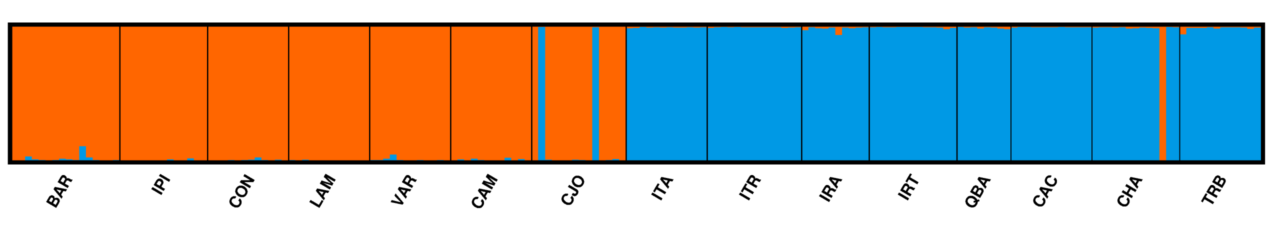


K=3

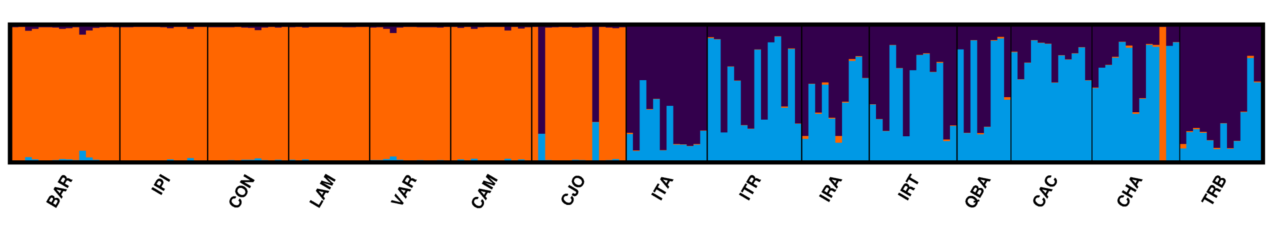


K=4

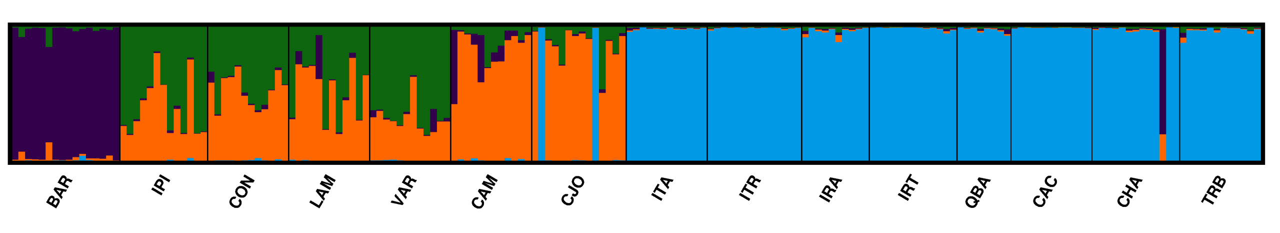


K=5

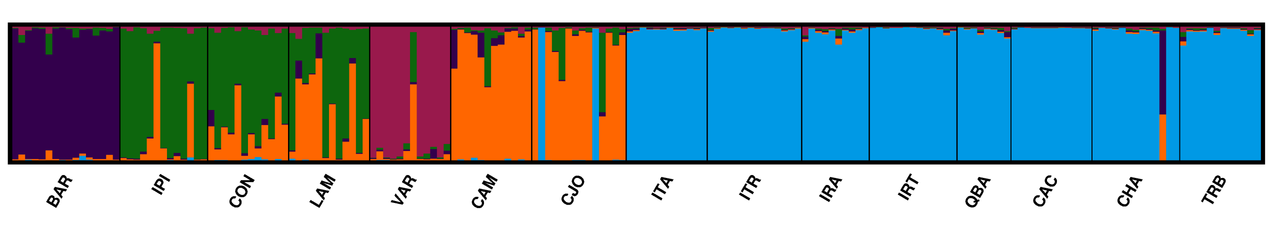


K=6

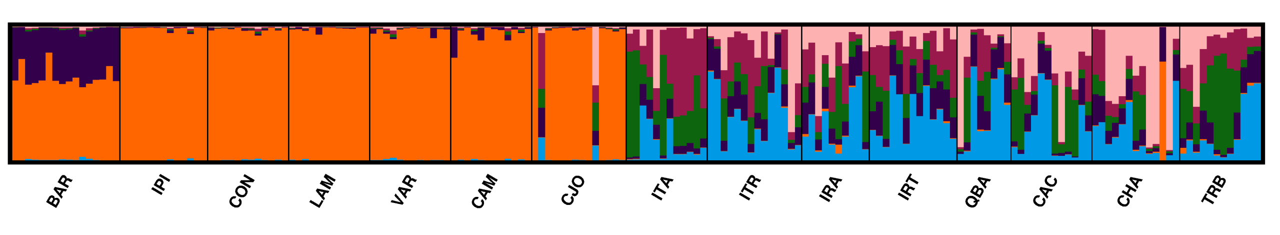


K=7

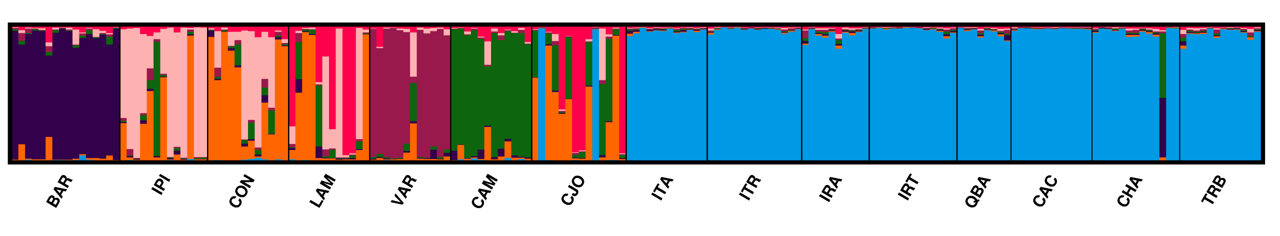


K=8

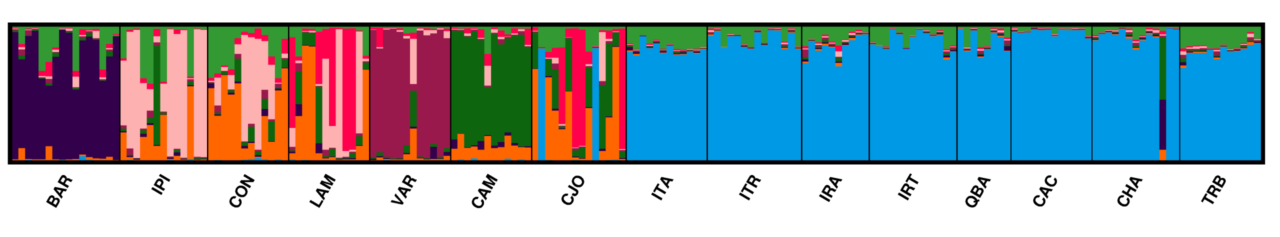


K=9

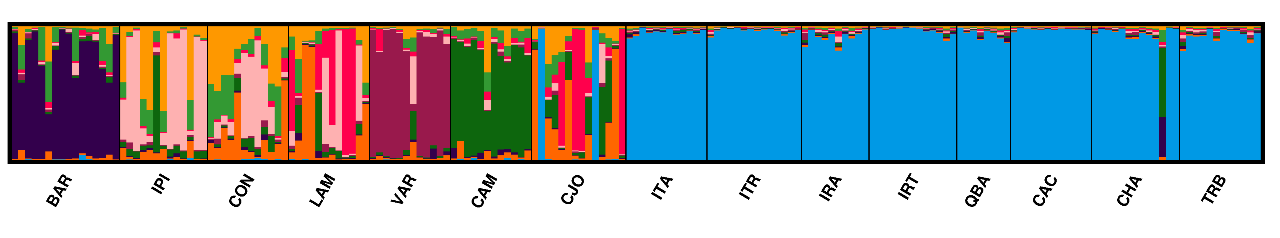


K=10

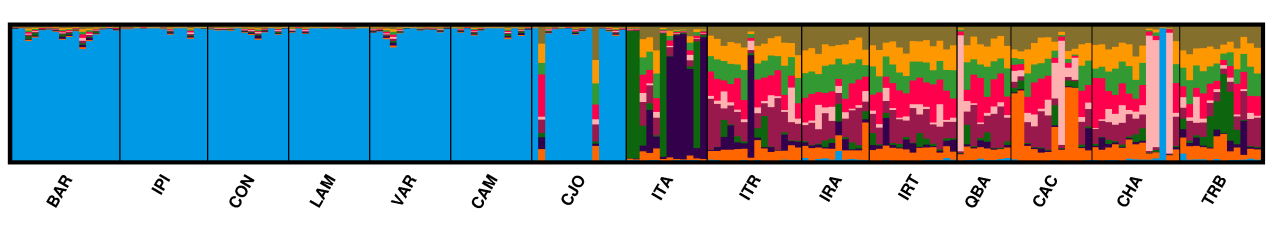


K=11

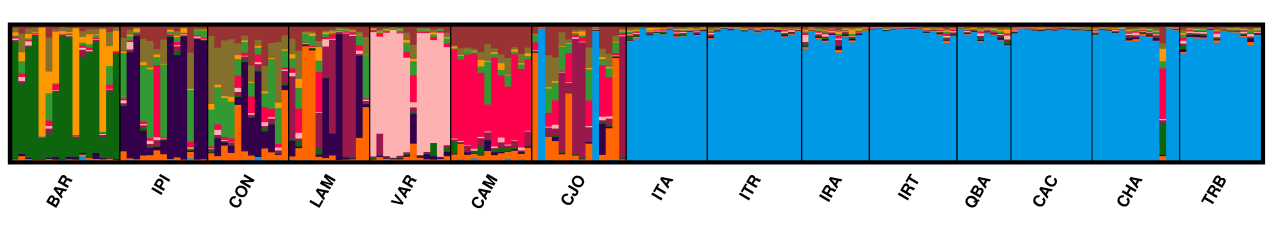


K=12

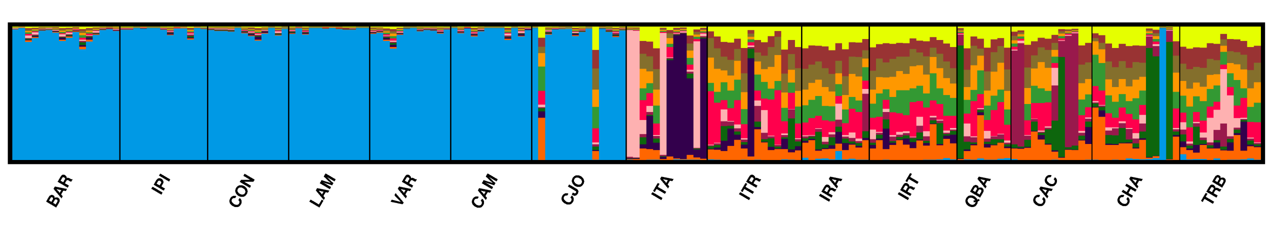


K=13

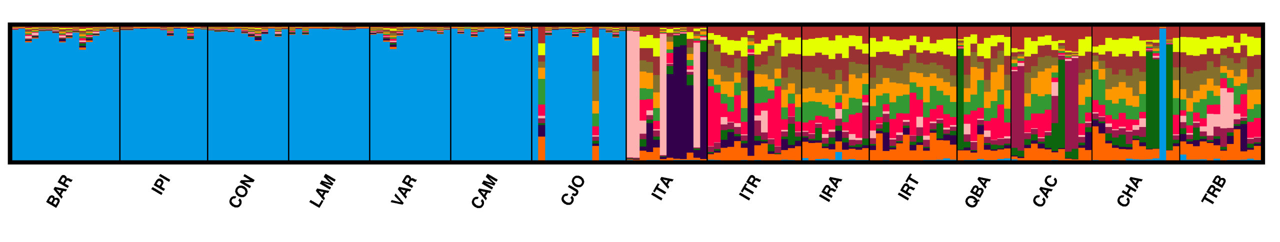


K=14

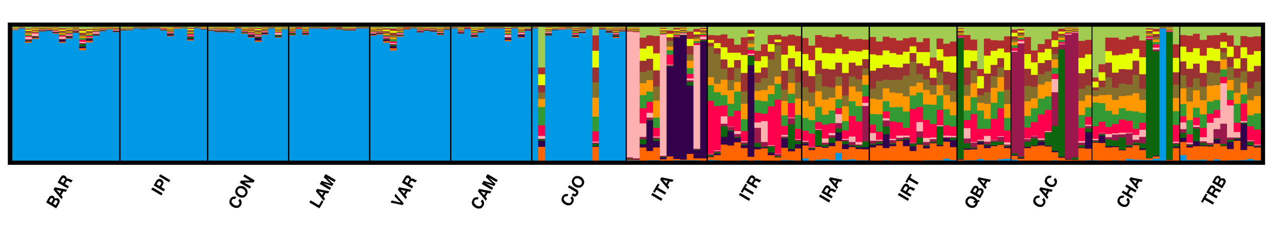


K=15

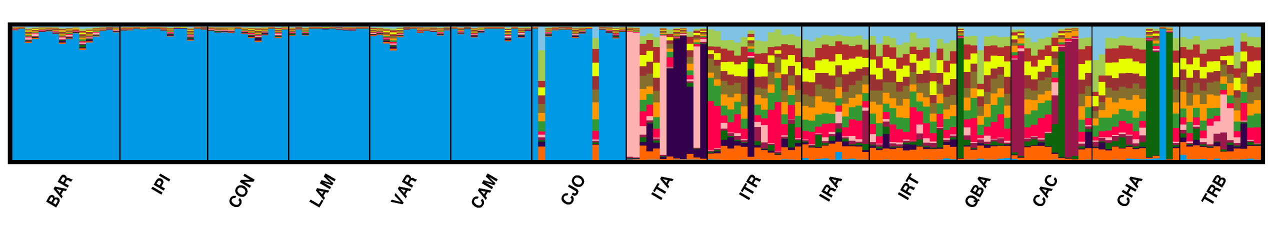


Minor modes for the uploaded data:

K=3 MinorCluster1

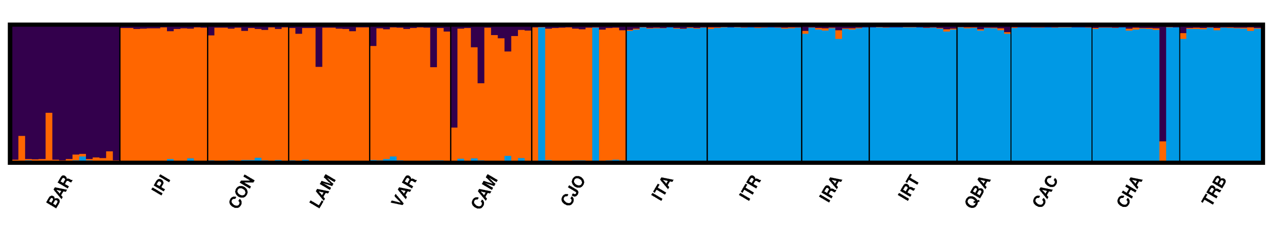


K=4 MinorCluster1

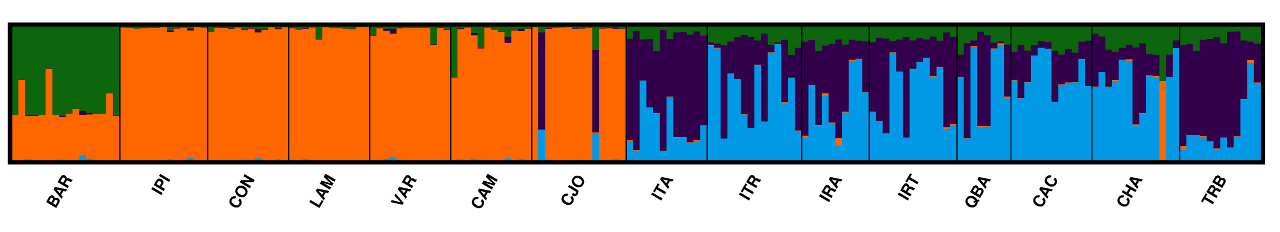


K=5 MinorCluster1

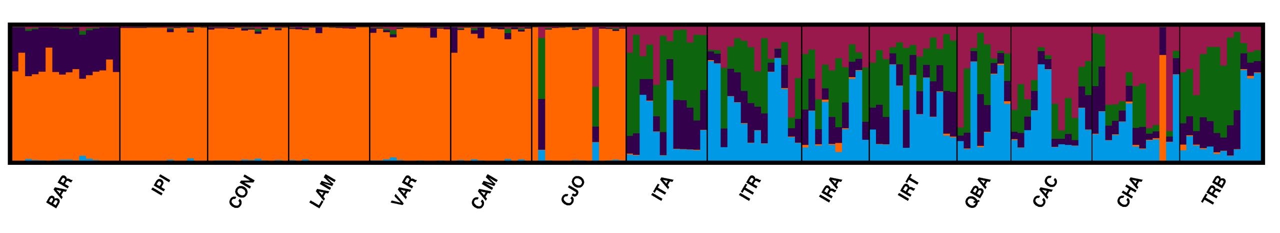


K=6 MinorCluster1

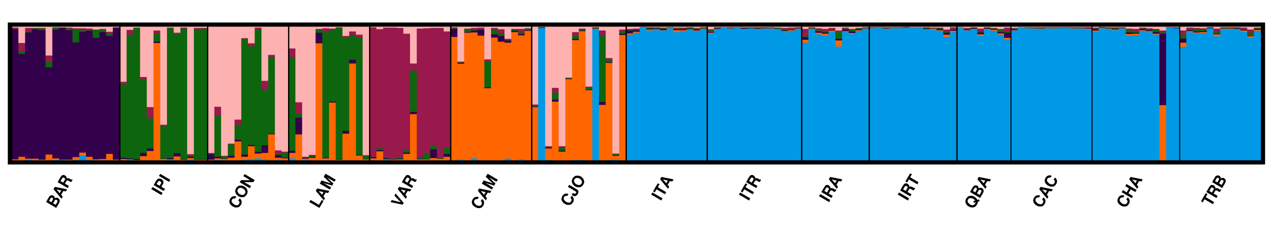


K=7 MinorCluster1

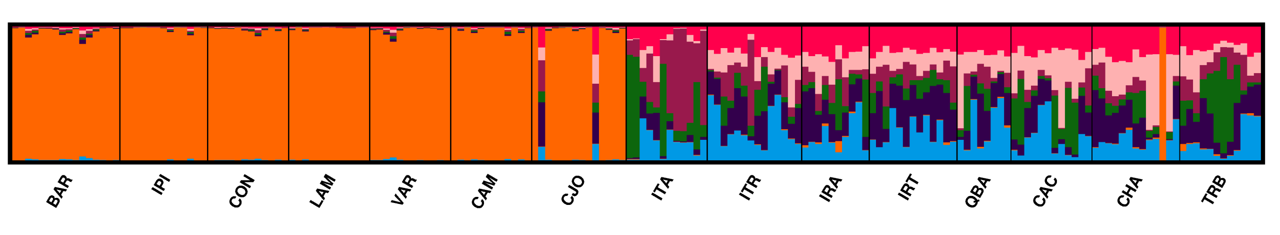


K=8 MinorCluster1

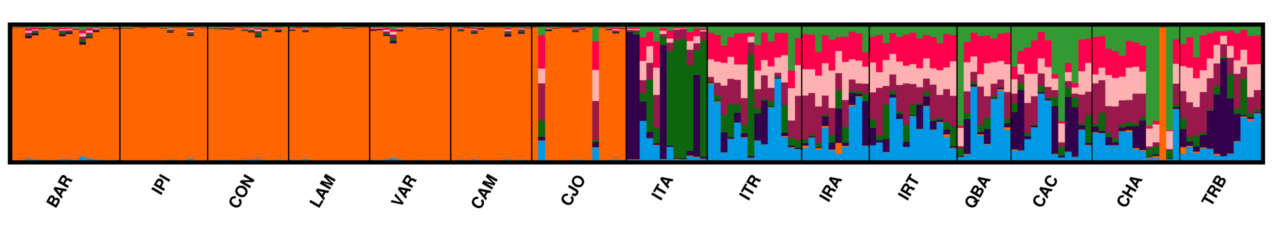


K=9 MinorCluster1

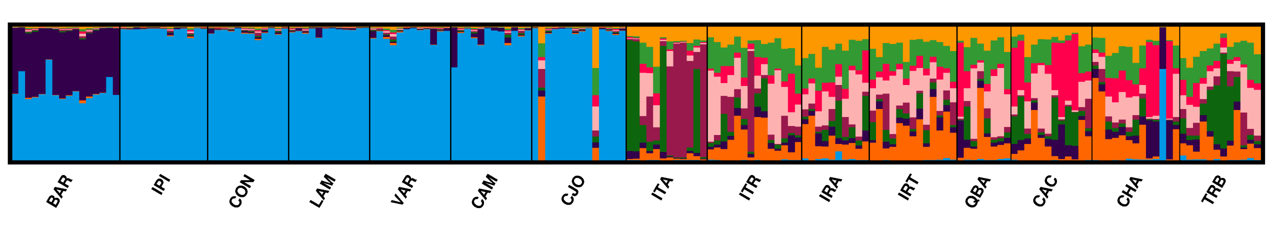


K=10 MinorCluster1

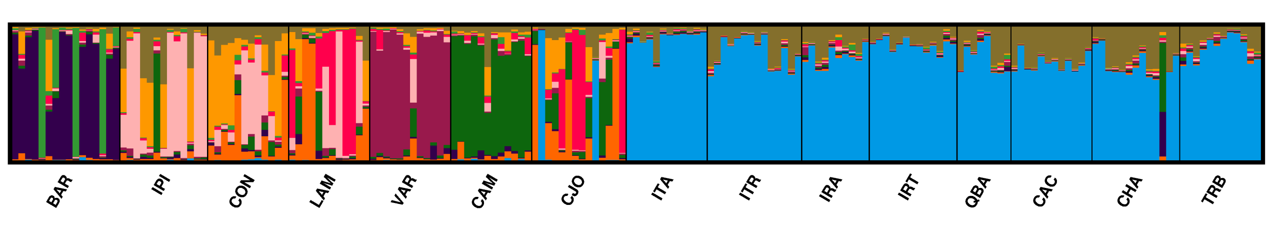


K=11 MinorCluster1

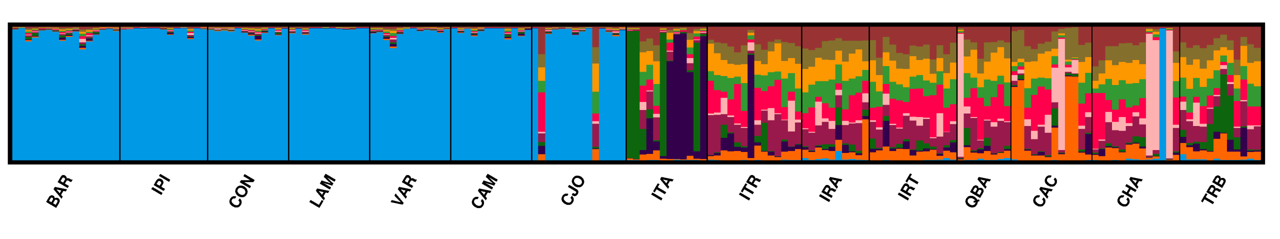


K=12 MinorCluster1

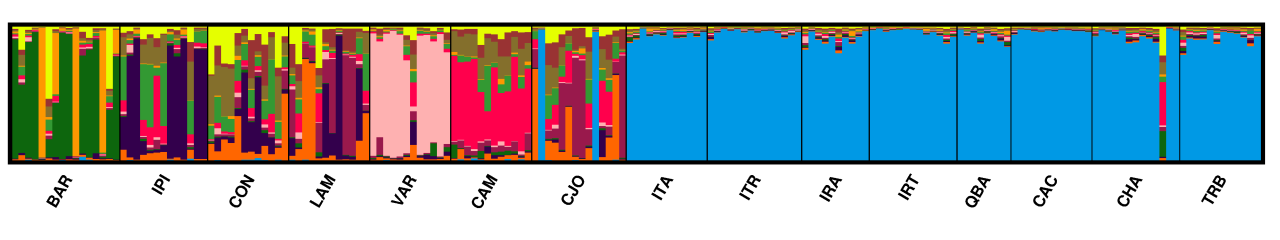


K=13 MinorCluster1

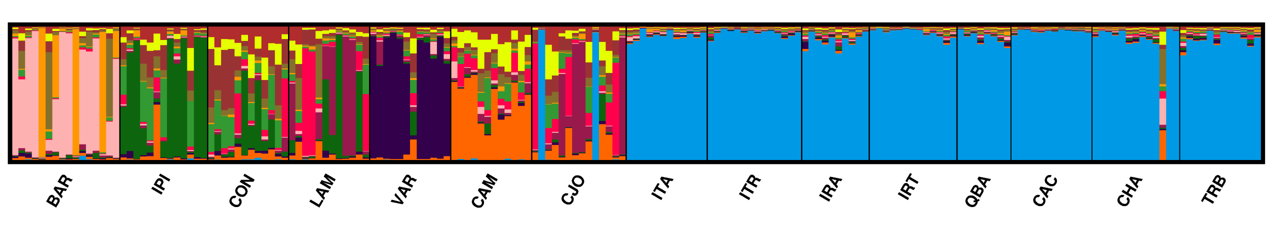


K=14 MinorCluster1

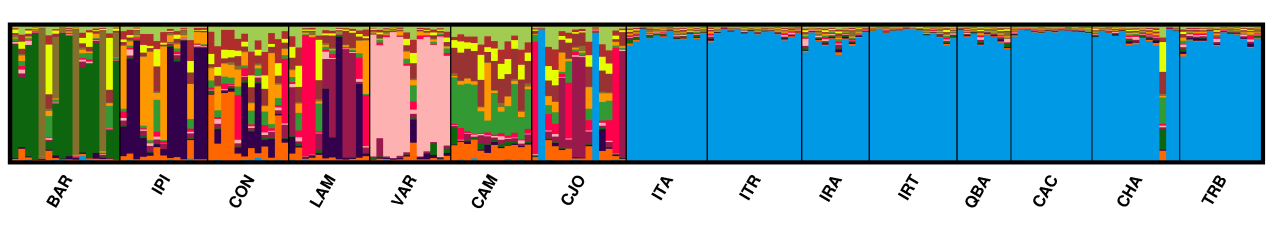


K=15 MinorCluster1

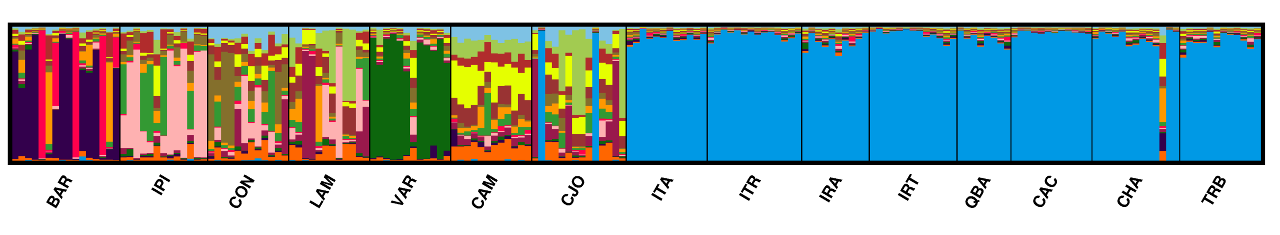


Division of runs by mode:

| | |
|---|---|
| K=1 | 8/8 |
| K=2 | 8/8 |
| K=3 | 6/8, 2/8 |
| K=4 | 5/8, 3/8 |
| K=5 | 5/8, 3/8 |
| K=6 | 5/8, 3/8 |
| K=7 | 4/8, 4/8 |
| K=8 | 6/8, 2/8 |
| K=9 | 4/8, 4/8 |
| K=10 | 5/8, 3/8 |
| K=11 | 6/8, 2/8 |
| K=12 | 5/8, 3/8 |
| K=13 | 5/8, 3/8 |
| K=14 | 5/8, 3/8 |
| K=15 | 5/8, 3/8 |
