# Supplementary material for: A 3K Axiom SNP array from a transcriptome-wide SNP resource sheds new light on the genetic diversity and structure of the iconic subtropical conifer tree Araucaria angustifolia (Bert.) Kuntze
Source: PLoS One. 2020 Aug 31;15(8):e0230404. doi: 10.1371/journal.pone.0230404 (PMC7458329; doi:10.1371/journal.pone.0230404)
Supplement: S8 File — (PDF) [file pone.0230404.s008.pdf]

CLUMPAK main pipeline - Job 1581706288 summary

Major modes for the uploaded data:

K=1

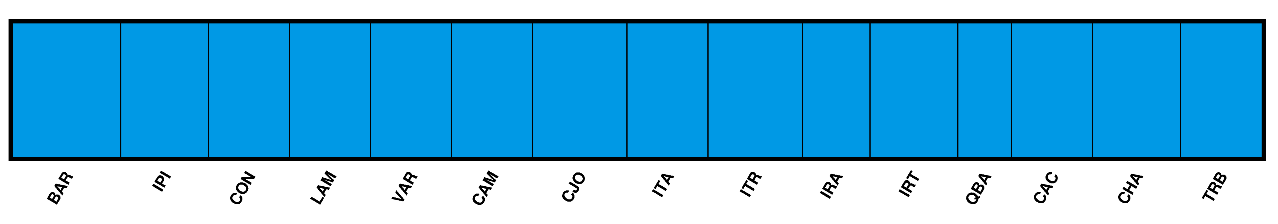

K=2

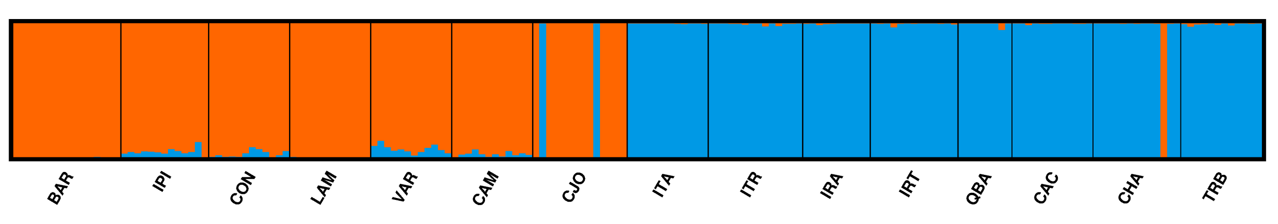

K=3

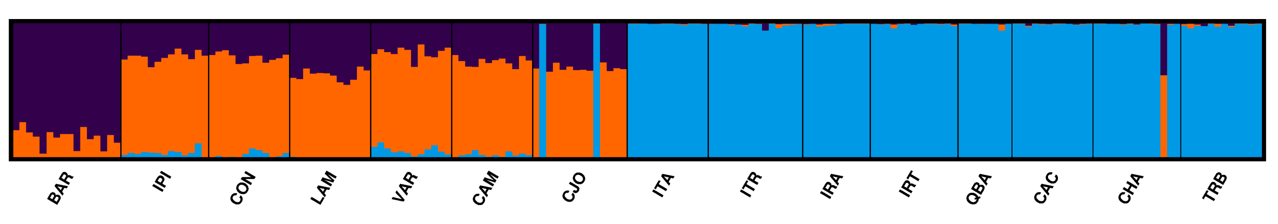

K=4

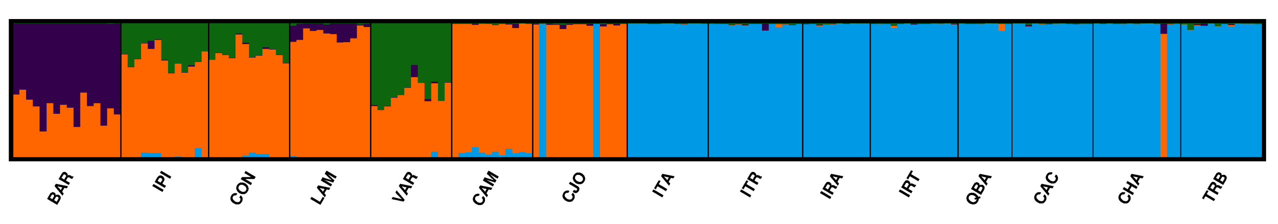

K=5

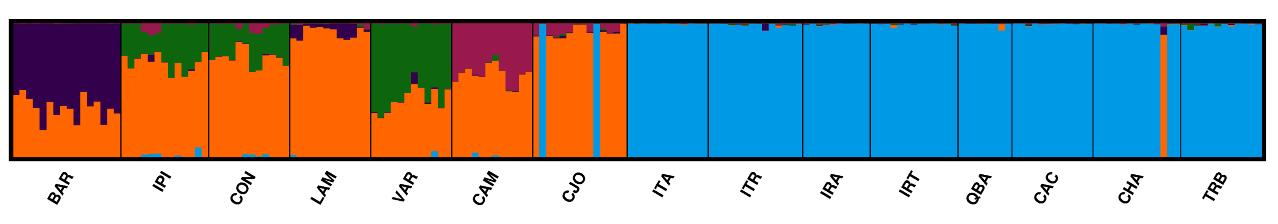

K=6

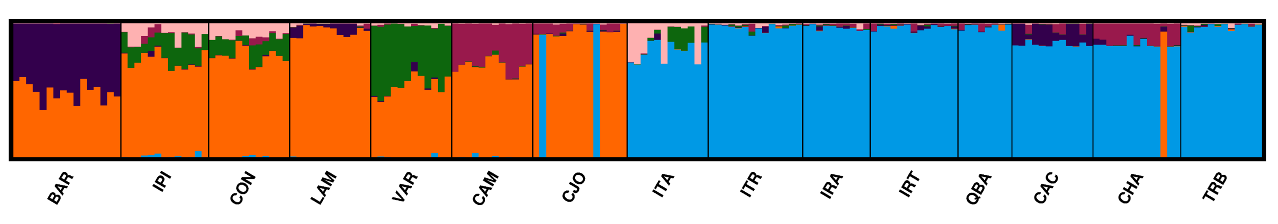

K=7

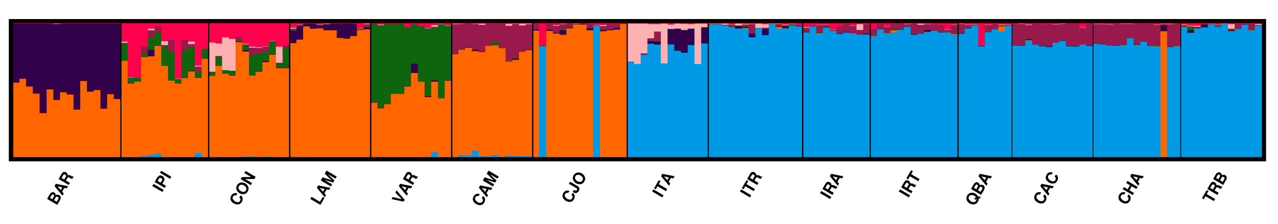

K=8

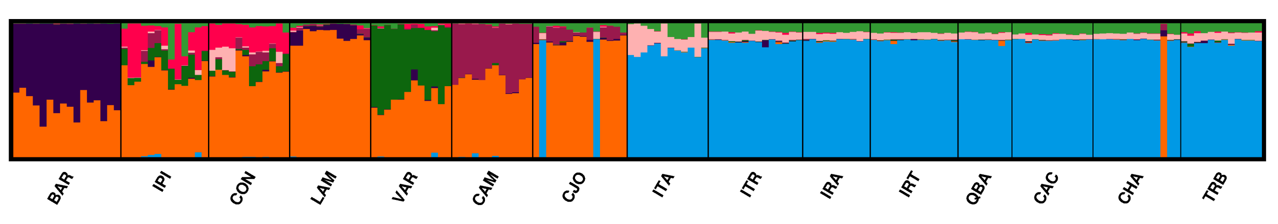

K=9

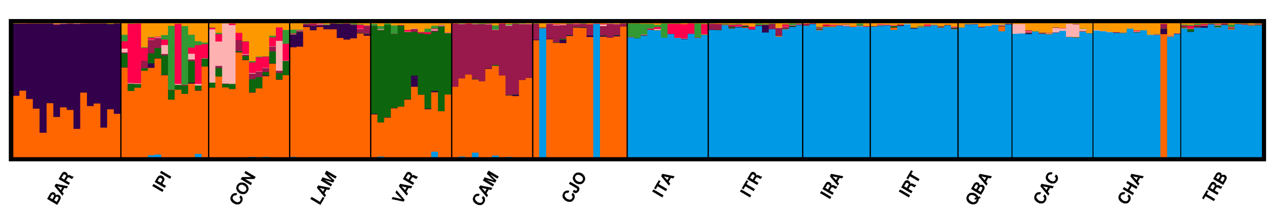

K=10

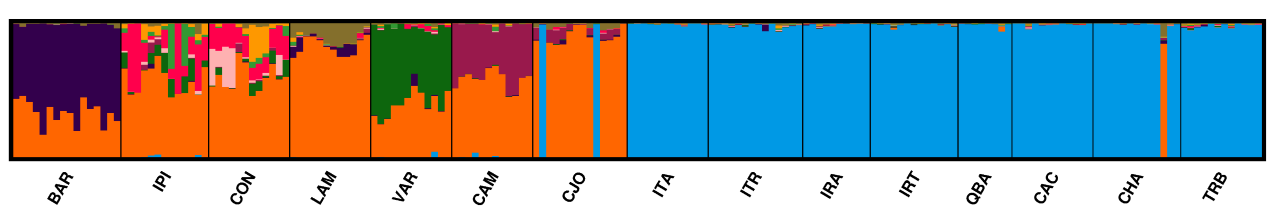

K=11

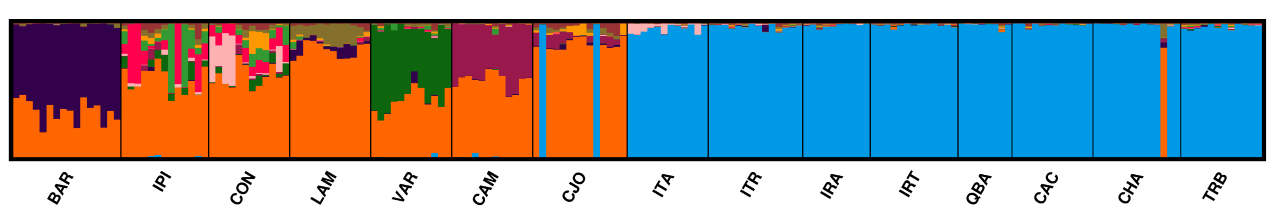

K=12

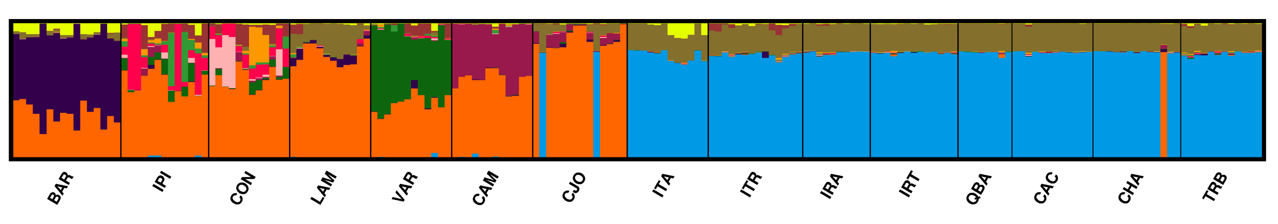

K=13

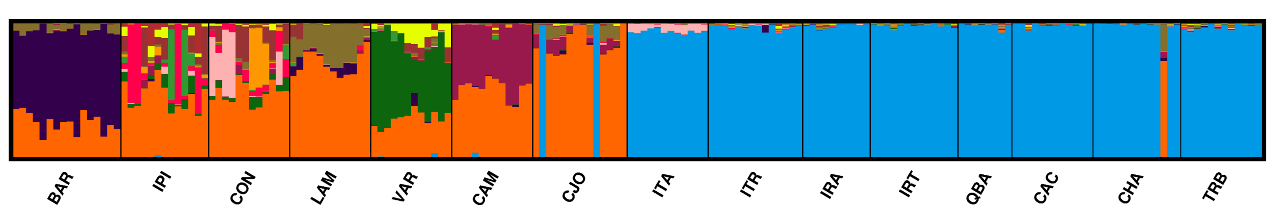

K=14

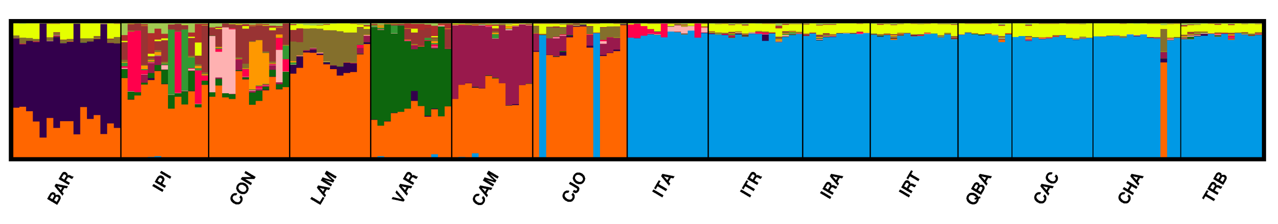

K=15

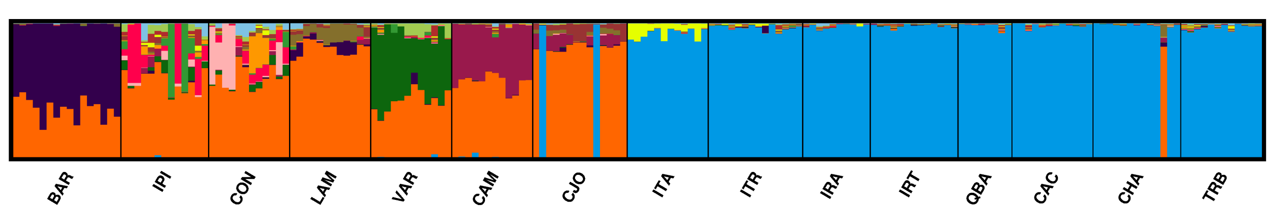

Minor modes for the uploaded data:

K=3 MinorCluster1

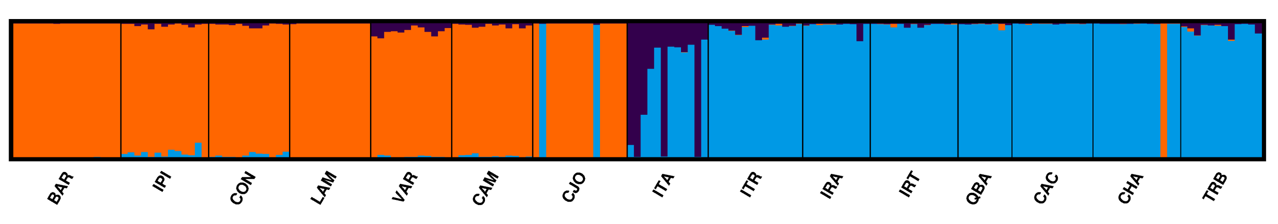

K=5 MinorCluster1

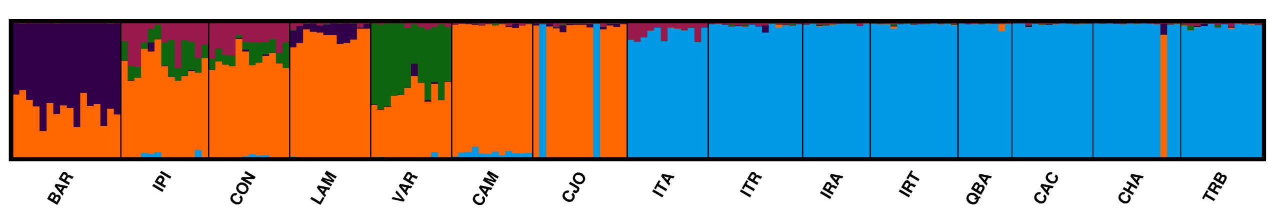

K=10 MinorCluster1

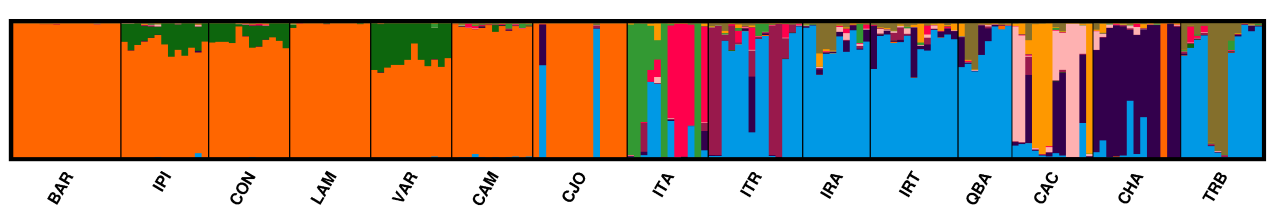

K=11 MinorCluster1

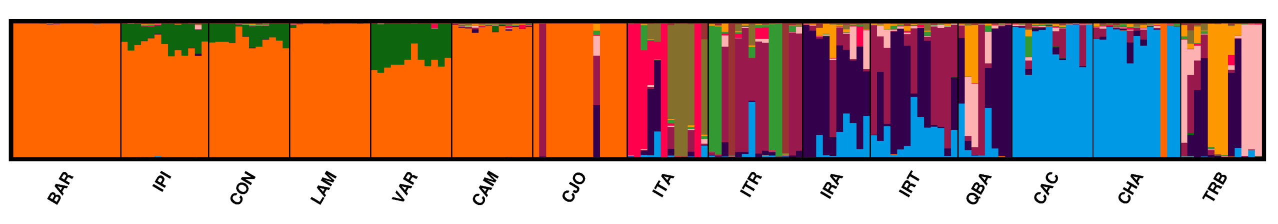

K=12 MinorCluster1

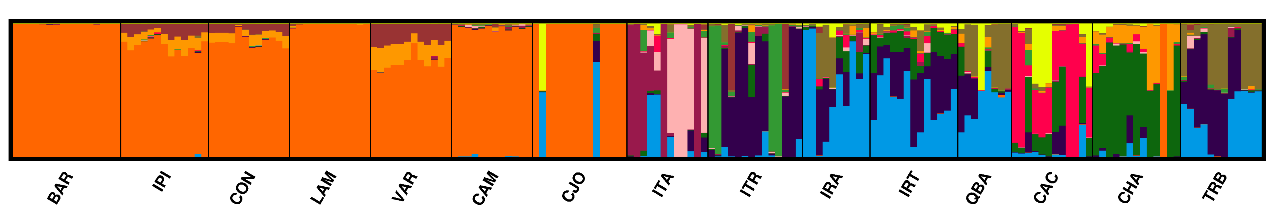

K=13 MinorCluster1

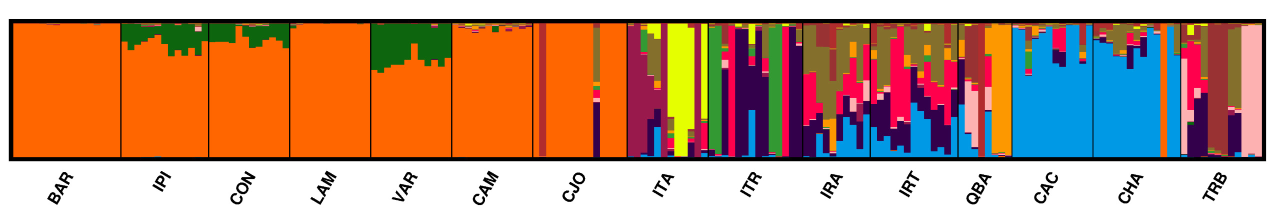

K=14 MinorCluster1

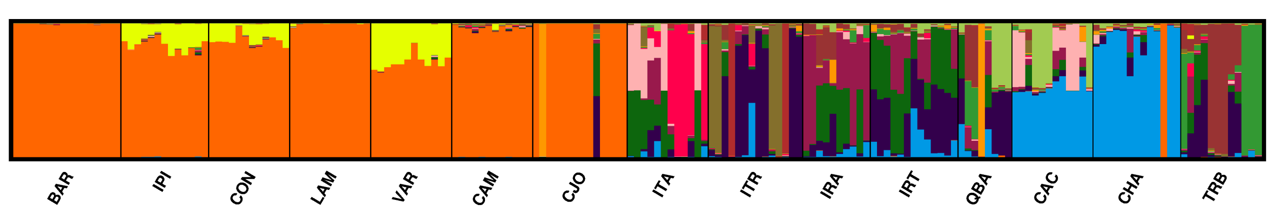

K=15 MinorCluster1

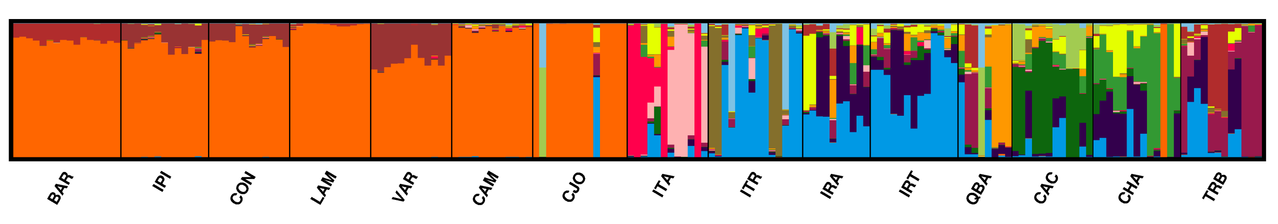

Division of runs by mode:

K=1	6/6
K=2	6/6
K=3	4/6, 2/6
K=4	6/6
K=5	3/6, 3/6
K=6	6/6
K=7	6/6
K=8	6/6
K=9	6/6
K=10	5/6, 1/6
K=11	5/6, 1/6
K=12	4/6, 2/6
K=13	4/6, 2/6
K=14	4/6, 2/6
K=15	3/6, 3/6
