# Supplementary material for: A 3K Axiom SNP array from a transcriptome-wide SNP resource sheds new light on the genetic diversity and structure of the iconic subtropical conifer tree Araucaria angustifolia (Bert.) Kuntze
Source: PLoS One. 2020 Aug 31;15(8):e0230404. doi: 10.1371/journal.pone.0230404 (PMC7458329; doi:10.1371/journal.pone.0230404)
Supplement: S1 Table — City and State of origin, number of samples genotyped, latitude, longitude and altitude of the populations sampled are provided. (DOC) [file pone.0230404.s009.doc]

**S1 Table.** Description of the genotyped samples from different populations of *Araucaria angustifolia* used to validate the 3K SNP Axiom® Array. City and State of origin, number of samples genotyped, latitude, longitude and altitude of the populations sampled are provided.

| Population | City/State of origin | CODE | Number of samples genotyped | Latitude | Longitude | Altitude  (m) |
| --- | --- | --- | --- | --- | --- | --- |
| 1 | Barbacena - MG | BAR | 16 | -21.000 | -43.833 | 1,206 |
| 2 | Ipiúna de Calda - MG | IPI | 13 | -21.667 | -46.167 | 1,300 |
| 3 | Congonhal - MG | CON | 12 | -21.700 | -46.250 | 854 |
| 4 | Lambarí - MG | LAM | 12 | -22.000 | -45.500 | 878 |
| 5 | Vargem Grande do Sul - SP | VAR | 12 | -21.500 | -46.500 | 800 |
| 6 | Camanducaia - MG | CAM | 12 | -25.500 | -50.600 | 880 |
| 7 | Campos do Jordão - SP | CJO | 14 | -19.000 | -45.500 | 1,600 |
| 8 | Itapeva - SP | ITA | 12 | -24.283 | -48.900 | 930 |
| 9 | Itararé - SP | ITR | 14 | -24.500 | -49.167 | 930 |
| 10 | Iratí - PR | IRA | 10 | -22.500 | -46.333 | 1,600 |
| 11 | Iratí (Tardio) - PR | IRT | 13 | -25.500 | -50.600 | 880 |
| 12 | Quatro Barras - PR | QBA | 8 | -25.333 | -59.233 | 915 |
| 13 | Caçador - SC | CAC | 12 | -26.767 | -51.017 | 960 |
| 14 | Chapecó - SC | CHA | 13 | -27.117 | -52.600 | 675 |
| 15 | Três Barras - SC | TRB | 12 | -25.250 | -50.300 | 760 |
