# Supplementary material for: A 3K Axiom SNP array from a transcriptome-wide SNP resource sheds new light on the genetic diversity and structure of the iconic subtropical conifer tree Araucaria angustifolia (Bert.) Kuntze
Source: PLoS One. 2020 Aug 31;15(8):e0230404. doi: 10.1371/journal.pone.0230404 (PMC7458329; doi:10.1371/journal.pone.0230404)
Supplement: S2 Table — Populations 7 and 8 in the transition zone display slightly differentiated Fst estimates from their regionally associated populations as indicated by the heatmap. All Fst estimates were significant (p<0.001) based on a permutation test by bootstrapping over loci. (DOC) [file pone.0230404.s010.doc]

**S2 Table.** Heat map of pairwise *F_st_* estimates based on 2,022 polymorphic SNPs among all 15 populations indicating the higher differentiation between the populations in the northern (1 to 7) and southern (8 to 15) regions, and lower differentiation between populations within regions. Populations 7 and 8 in the transition zone display slightly differentiated *F_st_* estimates from their regionally associated populations as indicated by the heatmap. All *F_st_* estimates were significant (p<0.001) based on a permutation test by bootstrapping over loci.

|  |  | **Northern populations** | | | | | | | **Southern populations** | | | | | | | |
| --- | --- | --- | --- | --- | --- | --- | --- | --- | --- | --- | --- | --- | --- | --- | --- | --- |
|  |  | **BAR** | **IPI** | **CON** | **LAM** | **VAR** | **CAM** | **CJO** | **ITA** | **ITR** | **IRA** | **IRT** | **QBA** | **CAC** | **CHA** | **TRB** |

| **Northern populations** | **BAR** | 0.000 |  |  |  |  |  |  |  |  |  |  |  |  |  |  |
| --- | --- | --- | --- | --- | --- | --- | --- | --- | --- | --- | --- | --- | --- | --- | --- | --- |
|  | **IPI** | 0.119 | 0.000 |  |  |  |  |  |  |  |  |  |  |  |  |  |
|  | **CON** | 0.118 | 0.022 | 0.000 |  |  |  |  |  |  |  |  |  |  |  |  |
|  | **LAM** | 0.105 | 0.054 | 0.056 | 0.000 |  |  |  |  |  |  |  |  |  |  |  |
|  | **VAR** | 0.150 | 0.060 | 0.069 | 0.104 | 0.000 |  |  |  |  |  |  |  |  |  |  |
|  | **CAM** | 0.137 | 0.073 | 0.070 | 0.077 | 0.114 | 0.000 |  |  |  |  |  |  |  |  |  |
|  | **CJO** | 0.093 | 0.032 | 0.034 | 0.032 | 0.073 | 0.045 | 0.000 |  |  |  |  |  |  |  |  |
| **Southern populations** | **ITA** | 0.378 | 0.329 | 0.341 | 0.365 | 0.343 | 0.354 | 0.284 | 0.000 |  |  |  |  |  |  |  |
|  | **ITR** | 0.345 | 0.300 | 0.311 | 0.335 | 0.314 | 0.328 | 0.256 | 0.044 | 0.000 |  |  |  |  |  |  |
|  | **IRA** | 0.365 | 0.315 | 0.327 | 0.353 | 0.330 | 0.344 | 0.268 | 0.045 | 0.023 | 0.000 |  |  |  |  |  |
|  | **IRT** | 0.348 | 0.303 | 0.313 | 0.337 | 0.314 | 0.328 | 0.257 | 0.045 | 0.015 | 0.011 | 0.000 |  |  |  |  |
|  | **QBA** | 0.365 | 0.314 | 0.326 | 0.352 | 0.331 | 0.344 | 0.262 | 0.052 | 0.021 | 0.014 | 0.011 | 0.000 |  |  |  |
|  | **CAC** | 0.368 | 0.319 | 0.332 | 0.357 | 0.335 | 0.350 | 0.276 | 0.066 | 0.039 | 0.031 | 0.024 | 0.031 | 0.000 |  |  |
|  | **CHA** | 0.326 | 0.280 | 0.290 | 0.311 | 0.293 | 0.304 | 0.234 | 0.057 | 0.031 | 0.025 | 0.018 | 0.023 | 0.026 | 0.000 |  |
|  | **TRB** | 0.352 | 0.304 | 0.317 | 0.342 | 0.318 | 0.332 | 0.260 | 0.046 | 0.021 | 0.017 | 0.015 | 0.018 | 0.040 | 0.033 | 0.000 |
