# Supplementary material for: A 3K Axiom SNP array from a transcriptome-wide SNP resource sheds new light on the genetic diversity and structure of the iconic subtropical conifer tree Araucaria angustifolia (Bert.) Kuntze
Source: PLoS One. 2020 Aug 31;15(8):e0230404. doi: 10.1371/journal.pone.0230404 (PMC7458329; doi:10.1371/journal.pone.0230404)

**S1 Fig.** Top 20 most abundant InterPro domains and families identified in the *A. angustifolia* non-redundant gene set**.** (A) InterPro domain annotation was performed on the non-redundant gene set obtained from the transcriptome assembly of *A. angustifolia*. (B) InterPro family annotation was performed on the non-redundant gene set obtained from the transcriptome assembly of *A. araucaria*.


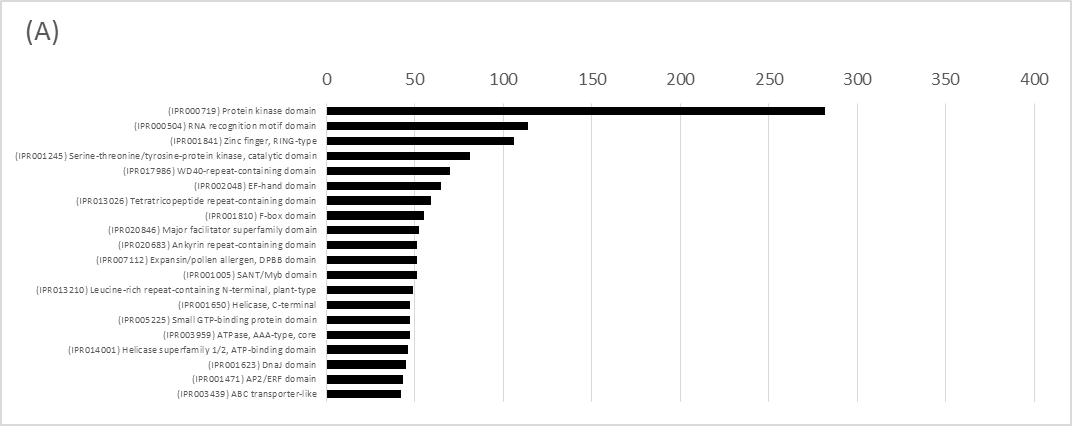


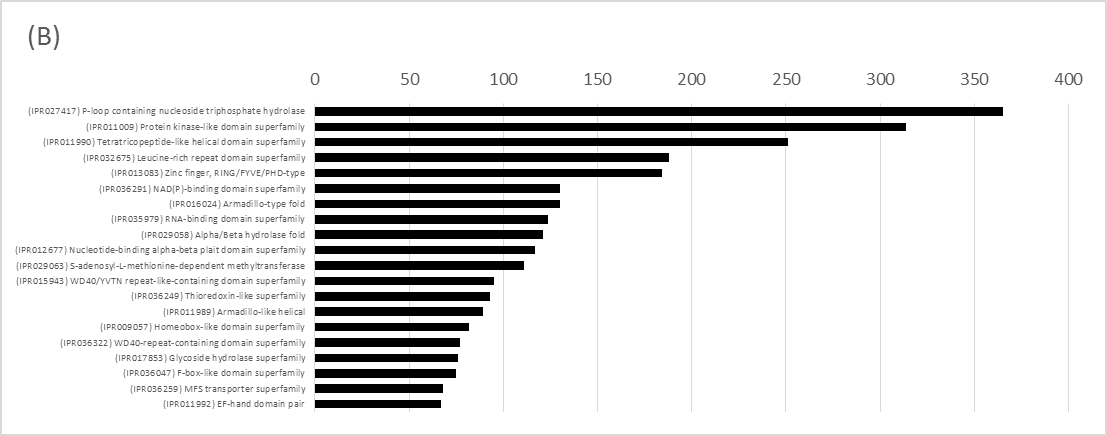

Supplement: S1 Fig — (A) InterPro domain annotation was performed on the non-redundant gene set obtained from the transcriptome assembly of A. angustifolia. (B) InterPro family annotation was performed on the non-redundant gene set obtained from the transcriptome assembly of A. araucaria. (DOC) [file pone.0230404.s011.doc]
