# Supplementary material for: A 3K Axiom SNP array from a transcriptome-wide SNP resource sheds new light on the genetic diversity and structure of the iconic subtropical conifer tree Araucaria angustifolia (Bert.) Kuntze
Source: PLoS One. 2020 Aug 31;15(8):e0230404. doi: 10.1371/journal.pone.0230404 (PMC7458329; doi:10.1371/journal.pone.0230404)

**S2 Fig.** Distribution of most abundant gene ontology (GO) terms in the three GO categories assigned to the *Araucaria angustifolia* contigs. Only level 3 terms are represented


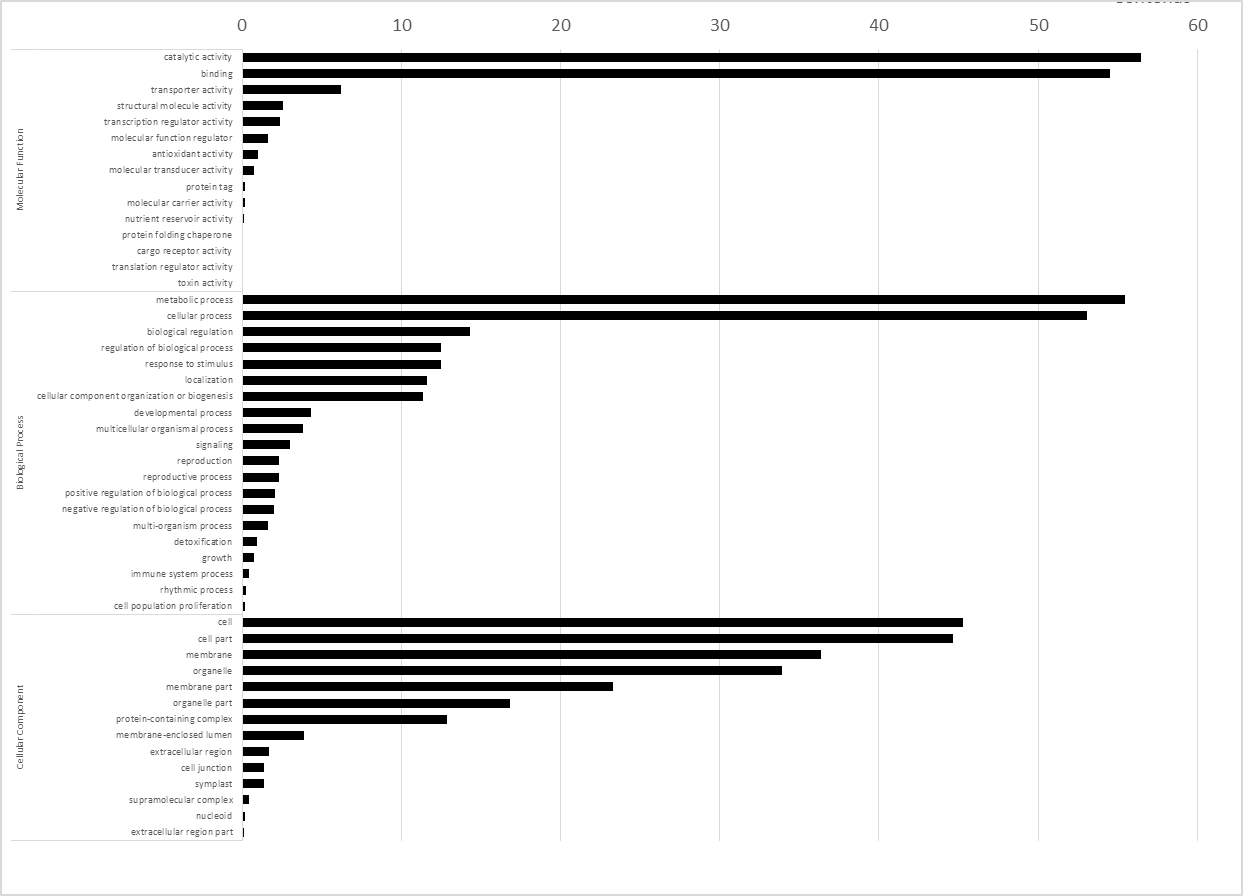

Supplement: S2 Fig — Only level 3 terms are represented. (DOC) [file pone.0230404.s012.doc]
