# Supplementary material for: A 3K Axiom SNP array from a transcriptome-wide SNP resource sheds new light on the genetic diversity and structure of the iconic subtropical conifer tree Araucaria angustifolia (Bert.) Kuntze
Source: PLoS One. 2020 Aug 31;15(8):e0230404. doi: 10.1371/journal.pone.0230404 (PMC7458329; doi:10.1371/journal.pone.0230404)

**S3 Fig.** SNP frequency spectrum of all 3,038 successfully genotyped SNPs by the two performance evaluation criteria. Also plotted are only the SNPs derived from RNA-seq data to show that the majority of monomorphic SNPs came from RAD-seq data.


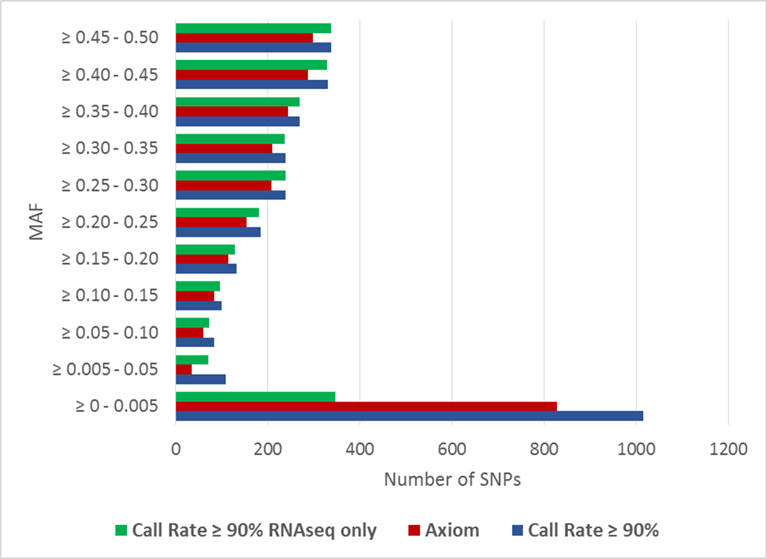

Supplement: S3 Fig — Also plotted are only the SNPs derived from RNA-seq data to show that the majority of monomorphic SNPs came from RAD-seq data. (DOC) [file pone.0230404.s013.doc]
