# Supplementary material for: A 3K Axiom SNP array from a transcriptome-wide SNP resource sheds new light on the genetic diversity and structure of the iconic subtropical conifer tree Araucaria angustifolia (Bert.) Kuntze
Source: PLoS One. 2020 Aug 31;15(8):e0230404. doi: 10.1371/journal.pone.0230404 (PMC7458329; doi:10.1371/journal.pone.0230404)

**S4 Fig.** Population structure analyses using a multidimensional Principal Coordinate analysis (PCoA) with the full set of 2,022 SNPs. Top panel: PCoA plot involving only the seven northern region populations; Bottom panel: PCoA plot involving only the eight southern region populations. The proportions of variation explained by the first three PCoA axes are indicated from left to right respectively in each plot.


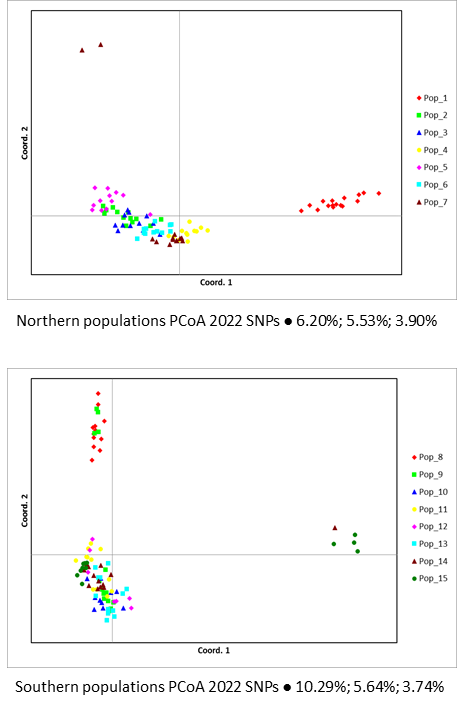

Supplement: S4 Fig — Top panel: PCoA plot involving only the seven northern region populations; Bottom panel: PCoA plot involving only the eight southern region populations. The proportions of variation explained by the first three PCoA axes are indicated from left to right respectively in each plot. (DOC) [file pone.0230404.s014.doc]
