# Supplementary material for: A 3K Axiom SNP array from a transcriptome-wide SNP resource sheds new light on the genetic diversity and structure of the iconic subtropical conifer tree Araucaria angustifolia (Bert.) Kuntze
Source: PLoS One. 2020 Aug 31;15(8):e0230404. doi: 10.1371/journal.pone.0230404 (PMC7458329; doi:10.1371/journal.pone.0230404)
Supplement: S5 Fig — (DOC) [file pone.0230404.s015.doc]

**S5 Fig.** Results of Evanno´s Delta K analysis to define the most probable number of populations with different sets of markers as indicated in the figure.

| **8 microsatellites**  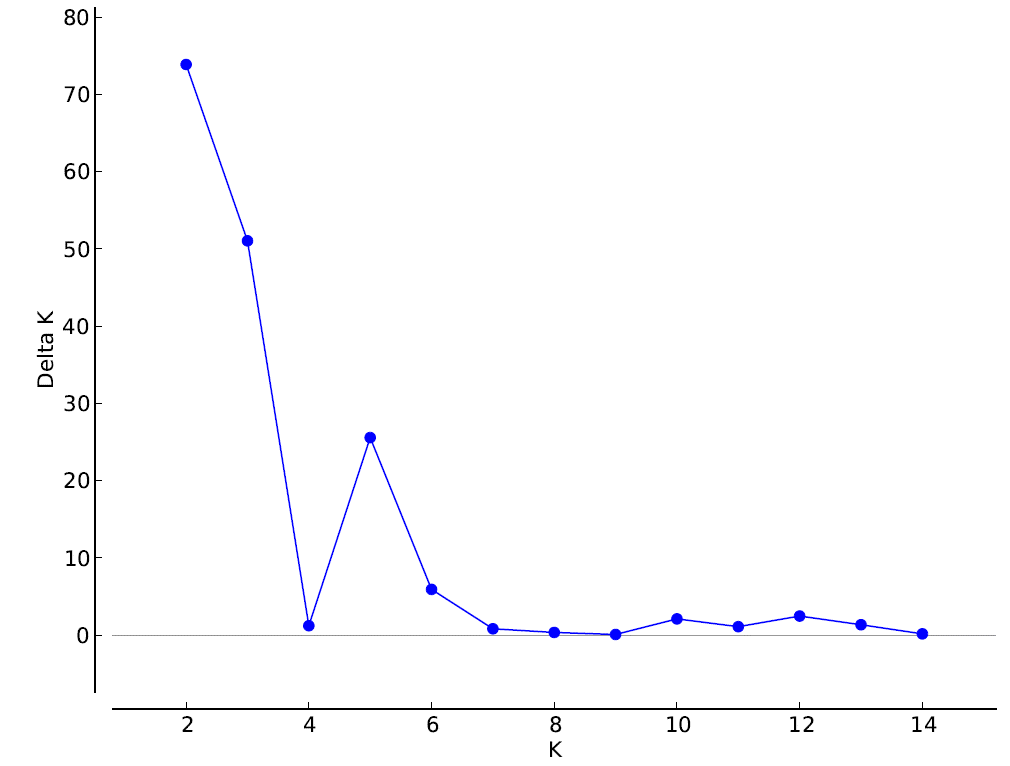 |
| --- |
| **80 SNPs**  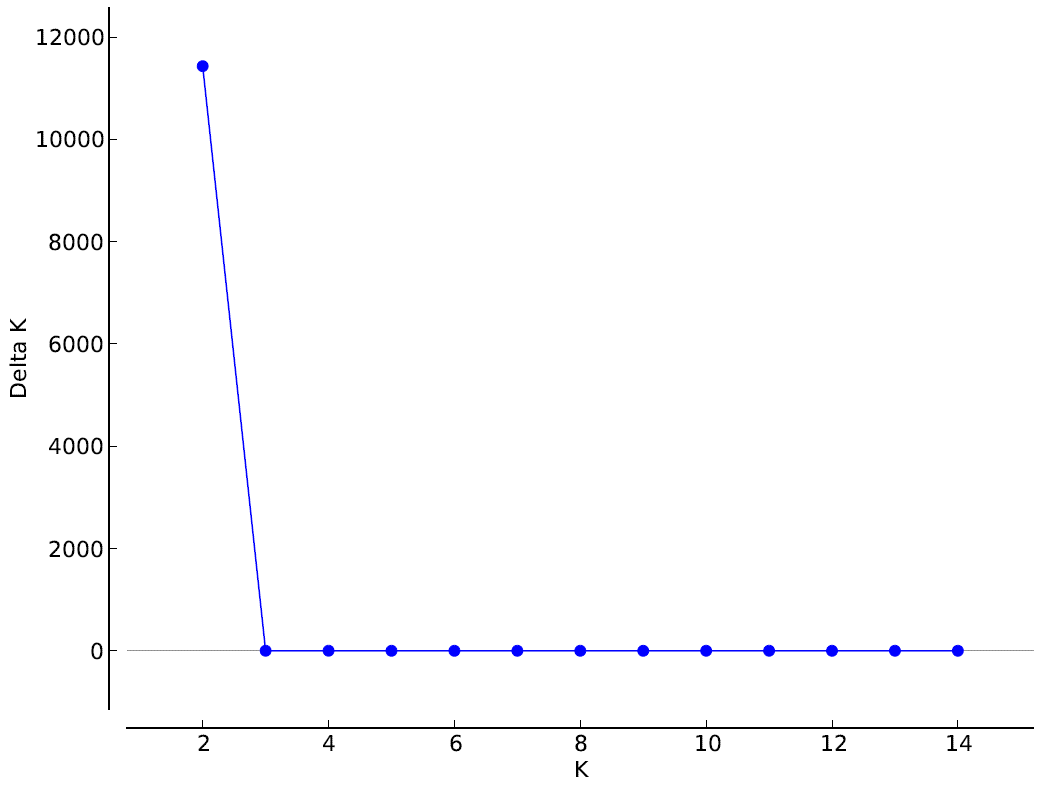 |
| **2,022 SNPs**  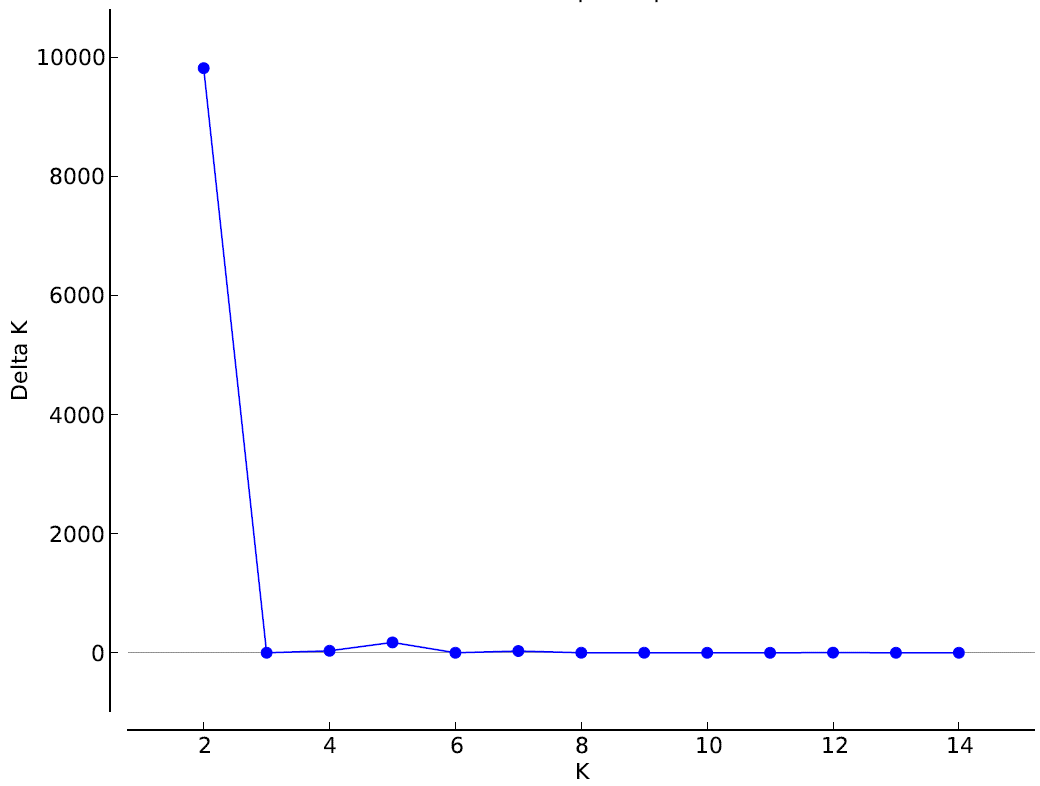 |
