# Supplementary material for: A 3K Axiom SNP array from a transcriptome-wide SNP resource sheds new light on the genetic diversity and structure of the iconic subtropical conifer tree Araucaria angustifolia (Bert.) Kuntze
Source: PLoS One. 2020 Aug 31;15(8):e0230404. doi: 10.1371/journal.pone.0230404 (PMC7458329; doi:10.1371/journal.pone.0230404)
Supplement: Attachment — Submitted filename: Response_to_reviewers_ PONE-D-20-05745_RevisedMS.pdf [file pone.0230404.s017.pdf]

RESPONSE TO EDITORIAL AND REVIEWERS REQUIREMENTS - PONE-D-20-05745

"A 3K Axiom® SNP array from a transcriptome-wide SNP resource sheds new light on the genetic diversity and structure of the iconic subtropical conifer tree Araucaria angustifolia (Bert.) Kuntze"

EDITORIAL REQUIREMENT

EDITOR: We understand that samples were taken from a species listed as Critically Endangered by the IUCN. In your Methods section, please provide additional information regarding the permits you obtained for the work. Please ensure you have included the full name of the authority that approved the field site access and, if no permits were required, a brief statement explaining why.

RESPONSE: Following the editorial requirements, we have included in the Methods section the full information regarding the governmental authorization we received to develop this research with *Araucaria angustifolia* being it a critically endangered species by the IUCN. The authorization number 02001.007609/2012-77 was issued by the Brazilian Institute of the Environment (IBAMA), the regulating body of the Brazilian Ministry of Environment in December 2012 giving full authorization to collect samples from natural populations for DNA analysis toward genetic studies

REVIEWER #1

REVIEWER #1: In the SNP filtering step, Line 221, the parameters of minimum allele frequency (MAF), missing rate, and Hardy-Weinberg equilibrium (HWE) were not used. The MAF filtering can exclude SNPs showing little variation in the population. The SNPs with a high missing rate indicated the number of detected genotype in the population is limited. The HWE filtering help exclude the SNPs arising from sequencing errors and natural selection. Therefore, these criteria are important for the selection of the SNPs.

RESPONSE: The section that the reviewer is indicating on line 221 does not refer to the filtering of SNPs following genotyping. Rather it describes the steps taken <u>during the SNP discovery process</u> using the VCF files of the RAD and RNA sequencing data or a few individuals used for that objective. The parameters taken during SNP discovery are evidently different ones based sequence context and parameters implemented by the GATK best practices. Also given that only a few individuals were sequenced for SNP discovery, neither MAF nor HWE would make any sense.

The parameters the reviewer is mentioning (MAF, call rate and HWE) were used <u>after SNP genotyping</u> is carried out with the array on population samples. This was done and fully explained in section " SNP array design and validation" on line 267. In that section these parameters were applied during the SNP validation process that followed the construction of the SNP array.

Still regarding the HWE filter, although this filter makes sense in human genetics or species that abide to the HWE expectations given a dioecious sexual reproduction, large populations and little or no structure, this filter does not apply to plant populations with mixed mating or selfing system in subdivided populations. For example, in selfing species, if a HWE filter is applied, all SNPs would be excluded as no or very few heterozygotes are seen. Additionally, in our case, since we genotyped individuals from several different populations, the consolidated set of data would never abide to HWE due to strong population differentiation that causes reduction n overall heterozygosity. So the HWE filter cannot be applied in our case as in essentially all plant population SNP arrays.

REVIEWER #1: In Line 285, 294, 408, MAF ≥ 0.005 was used to declare a polymorphic SNP. However, the commonly recognized and used MAF threshold is 0.05 or 0.01 for valid SNPs. MAF ≥ 0.005 indicated that the array may contain a number of rare SNPs. A MAF distribution plot should be provided to help evaluate this issue.

RESPONSE: We do acknowledge that commonly the MAF threshold used is MAF> 0.05. However this is really a threshold that depends on the sample size used and the envisaged applications of the SNP array. As we describe in detail in that same section " SNP array design and validation" we used a call rate ≥ 97% or ≥ 90% depending on the criteria adopted and MAF ≥ 0.005 for two reasons. We did deliberately intend to include rare SNPs in our array as these are in fact informative especially if one intends to use the SNP array for association analysis.

Low-frequency and rare variants have been shown to represent an important and understudied component of complex trait genetics. The literature on the importance of rare variants is abundant especially in human genetics (see for example doi: 10.1126/science.1219240 and doi: 10.1007/978-1-4939-7868-7_5) and methods have been increasingly been developed and used to deal with rare variants in GWAS (see for example doi: 10.1002/cphg.83). The second reason is that we genotyped different Araucaria populations and SNP allele frequencies vary across populations such that a SNP may be rare in one population and frequent in another. This is commonly seen in plant populations across large geographical areas mainly due to drift and occasionally selection. So the information content of SNPs will vary across populations and this fully justifies adopting a much lower MAF threshold to include all SNPs with such behavior. The genotyping quality of the SNPs was evaluated by the dish quality parameter that has to do with the quality of cluster separation among other things.

Regarding a MAF distribution plot, which we called site frequency spectrum (SFS) plot, it was adequately provided this in our supplementary material, Figure S3 and cited in the manuscript on line 427. As can be seen in the figure, the SFS does in fact show an enrichment on rare variants. As explained above, this was fully intentional and justified. However the plot also shows enrichment toward higher frequency SNPs.

REVIEWER #1: In Line 271, the author mentioned that the 3K SNP probes for the conifer were tilled on a multispecies Axiom® myDesign ™ array that contained a total of 51,867 SNPs, shared among five different plant species. What is the phylogenetic relationship of these species and more importantly, the genetic divergence of these species? If they are closely related species, some SNPs may inter-influence and cause false genotyping result.

RESPONSE: We would like to thank the reviewer for this question, which provides an opportunity to explain what we did. We added a sentence in Methods explaining this issue, to better clarify the readership.

The development of this multispecies array will be the topic of a specific methods publication under preparation. For right now we presented it as an invited talk at Plant Genome which can be viewed in Youtube (https://www.youtube.com/watch?v=-Wfb6WftQiU), and the reference was cited in the manuscript (reference 62). The five species were Araucaria angustifolia (Brazilian pine – order Pinales), Anacardium occidentale (cashew – order Sapindales), Manihot esculenta (cassava – order Malpighiales), Coffea robusta (coffee – order Gentianales) and Eucalyptus sp. (order Myrtales). These species are phylogenetically quite distant. Not only they belong to different families but also to different phylogenetic orders. Additionally, during array design all 51 thousand plus probes designed were subject to a detailed bioinformatics analysis to avoid probe homology such that SNP probe cross talking was precluded. Finally, even in the unlikely case that some cross talk signal happens, an analytical step can be used in Axiom Suite to quench the fluorescent signal of unwanted probes such that only the signal of the probes of the species under analysis is captured. Reminding, evidently, that only a single species sample is hybridized to the array at the time, in other words

no DNA mixing is used. So we are fully confident that no false genotyping result happened or will happen for any of the five species.
