# Supplementary material for: A 3K Axiom SNP array from a transcriptome-wide SNP resource sheds new light on the genetic diversity and structure of the iconic subtropical conifer tree Araucaria angustifolia (Bert.) Kuntze
Source: PLoS One. 2020 Aug 31;15(8):e0230404. doi: 10.1371/journal.pone.0230404 (PMC7458329; doi:10.1371/journal.pone.0230404)
Supplement: Attachment — Submitted filename: reviewer opinions.docx [file pone.0230404.s016.docx]

This manuscript reported a 3k SNP array for the conifer tree *Araucaria angustifolia* and its utility in genetic structure and diversity analysis. The SNP array was developed based on transcriptome sequencing data and pooled RAD-seq data，and had a better performance in genetic diversity and population structure analyses, compared to microsatellite markers.

However, I have a number of major concerns that I think need addressed prior to consideration for publication.

1. In the SNP filtering step, Line 221, the parameters of minimum allele frequency (MAF), missing rate, and Hardy-Weinberg equilibrium (HWE) were not used. The MAF filtering can exclude SNPs showing little variation in the population. The SNPs with a high missing rate indicated the number of detected genotype in the population is limited. The HWE filtering help exclude the SNPs arising from sequencing errors and natural selection. Therefore, these criteria are important for the selection of the SNPs.
2. In Line 285, 294, 408, MAF ≥ 0.005 was used to declare a polymorphic SNP. However, the commonly recognized and used MAF threshold is 0.05 or 0.01 for valid SNPs. MAF ≥ 0.005 indicated that the array may contain a number of rare SNPs. A MAF distribution plot should be provided to help evaluate this issue.
3. In Line 271, the author mentioned that the 3K SNP probes for the conifer were tilled on a multispecies Axiom® myDesign ™ array that contained a total of 51,867 SNPs, shared among five different plant species. What is the phylogenetic relationship of these species and more importantly, the genetic divergence of these species? If they are closely related species, some SNPs may inter-influence and cause false genotyping result.
