# Supplementary material for: A 3K Axiom SNP array from a transcriptome-wide SNP resource sheds new light on the genetic diversity and structure of the iconic subtropical conifer tree Araucaria angustifolia (Bert.) Kuntze
Source: PLoS One. 2020 Aug 31;15(8):e0230404. doi: 10.1371/journal.pone.0230404 (PMC7458329; doi:10.1371/journal.pone.0230404)
Supplement: Attachment — Submitted filename: Response_to_reviewers_ PONE-D-20-05745_R1_RevisedMS.docx [file pone.0230404.s018.docx]

**RESPONSE TO ADDITIONAL REVIEWERS REQUIREMENTS - PONE-D-20-05745R1**

*“A 3K Axiom® SNP array from a transcriptome-wide SNP resource sheds new light on the genetic diversity and structure of the iconic subtropical conifer tree Araucaria angustifolia (Bert.) Kuntze”*

**REVIEWER #1**

**REVIEWER #1 COMMENT: For the minor allele frequency, the authors have raised two reasons for the selection of 0.005. However, since the total number of the SNPs tilling on the array is only 3k, the rare SNPs are difficult to represent significance in both GWAS and in different populations. I still think a higher filtering parameter for MAF is necessary to ensure a higher genotyping rate.**

**RESPONSE:** As we had responded previously, the threshold used was in fact justified by the two reasons we outlined before. On one hand we deliberately intended to include rare SNPs in our array as these could be informative in specific situations. However the strongest justification was that the estimates were obtained in limited samples sizes for each one of several populations, and in light of the strong differentiation detected, it is likely that SNP allele frequencies vary across populations, such that a SNP allele may be rare overall but it may have higher frequency in some specific populations and thus informative. So, these rare alleles could in fact be well represented in some populations when larger samples sizes are genotyped. This is commonly seen in outbred plant populations across large geographical areas mainly due to drift and occasionally selection.

Regarding the second point we do agree with the reviewer that to include rarer SNPs it is important to ensure high genotyping rate. As described in the MS, we did use stringent dish quality and call rates metrics supported by the Axiom technology (Call rate > 97% for Axiom criteria) such that the quality of the genotype call is robust, despite the low estimated MAF. So we really don’t see any reason to exclude these seemingly rare SNPs from our evaluation in this initial array performance. Certainly as larger samples are studied across variable populations we are confident that several of these rarer SNPs will display higher frequencies.

Another point that should be mentioned is that by including rarer SNPs on the array, the ascertainment bias toward having largely frequent SNPs typical seen in array design, gets mitigated somehow adding to the value of the array. We had discussed this point in the MS.

In any case to address the reviewer’s concern, we added a paragraph to the MS, based on our arguments above, further detailing the reasons why we used a lower MAF threshold. We are confident that this would clarify the readership about our decision. We wrote:

*“Despite the fact that typically only SNPs with MAF ≥ 0.05 are considered as polymorphic, we deliberately intended to take into account rare SNPs in our array for two reasons: (1) rare SNPs could be informative for some specific applications such as gene flow, parentage and association studies; (2) we used stringent dish quality and call rates metrics criteria such that the quality of the genotype calls are robust, despite the low MAF; and (3) by having genotyped limited samples of individuals in this first study for each one of several populations, and in light of the strong differentiation detected (see below), it is expected that SNP allele frequencies will vary across populations, such that a SNP allele may be rare overall but it may have higher frequency and thus be informative in specific populations. So, these rare SNP alleles could turn out to be well represented in some populations when larger samples sizes are genotyped, especially for outbred forest tree populations across large geographical areas.”*

**REVIEWER #2**

Minor comments

**REVIEWER #2 COMMENT 1. Line 60, “ low to ultra-low sequencing of the whole genome.” what is low sequencing? Do you mean “low-coverage” or “low-cost”?**

**RESPONSE:** Thanks. We really mentioned low-coverage sequencing which also tend to be low-cost from a general point of view. We fixed that.

**REVIEWER #2 COMMENT 2. line 78, I disagree with this comment. I think that in addition to humans and animals, many forest trees already have high-density SNP chips for years, including conifers (Mol Ecol Resour. 2013;13(2):324–36. BMC Genomics, 2020;21:9)**

**RESPONSE:** Yes we agree and we added forest trees to the sentence. Additionally, as can be read a few paragraphs down the introduction, we had fully cited all the conifer chips published up to the time we had submitted the MS, including the Mol Ecol Res 2013 reference mentioned. We have now added the Howe et al. 2020 reference to the citations to make it fully complete.

**REVIEWER #2 COMMENT 3. line 168, the StringTie was used for transcriptome assembly, I think this tool need BAM format data that aligned reads to genome as input, so any references genome was available in this study?**

**RESPONSE:** Thank you for this opportunity to better clarify our methods in the manuscript. In fact, we have not used StringTie for the transcriptome assembly. As described in the prior MS version, we have adopted the StringTie approach only as an attempt to reconstruct cDNA fragments for both ends of the reads from the RNA-Seq data. The reviewer is correct to notice that StringTie uses BAM formatted data as input and also requires reads to be aligned to a reference genome. However, the StringTie tool includes a separate routine called SR (from Super-Reads), in which the input is constituted of unaligned paired-end reads. This is an optional step for preparing the alignments for the input of StringTie+SR runs (described in the StringTie manuscript, (https://www.ncbi.nlm.nih.gov/pmc/articles/PMC4643835/).

StringTie+SR borrows some algorithmic techniques from de novo genome assembly (i.e, do not need an available reference genome) to help with transcript assembly. Its input can include longer contigs that it assembles de novo from unambiguous, non-branching parts of a transcript. If the RNA-Seq data is paired, it is possible to use the SR routine to reconstruct the RNA-Seq fragments from their end sequences, which the authors of the tool have called Super-Reads (please see the StringTie+SR manual,

<http://ccb.jhu.edu/software/stringtie/index.shtml?t=manual#assembly>).

It can be used as an optional step for preparing these longer contigs for the input of StringTie+SR or any other transcriptome assembly tool that can handle single-end longer contigs that correspond approximately to the original cDNA fragment (such as an EST assembler tool). Because the StringTie+SR still relies on the alignment of these Super-Reads on the top of an reference genome, which is not available in the case of the Araucaria angustifolia, we just use this data as an input of the miraSearchESTSNPs assembler as described later in the text. To further clarify these points, we rewrote this section in the manuscript as follows:

*“As a first step, we used the StringTie+SR [51] method to pre-assemble longer contigs from the RNA-Seq paired-end data aiming to achieve the better performance of the subsequent transcriptome assembly. From StringTie+SR, we ran the perl script SR (Link to external file: http://ccb.jhu.edu/software/stringtie/dl/superreads.pl) separately on the reads of each three megastrobiles and each three somatic	embryogenic	cultures	of A. angustifolia. The SR method pre-assembled de novo the paired-end reads from the unambiguous, non-branching parts of the transcript in these six sources of cDNA data, resulting in super-reads of approximately 300 bp corresponding to the entire sequence of the original cDNA fragment. These super-reads were used as input of the miraSearchESTSNPs from MIRA 4 assembler [52] to generate the consensus sequences of the transcript assembly from the different sources of cDNA and to perform the SNP detection within this assembly.”*

**REVIEWER #2 COMMENT 4. It is true that SNP will definitely outperform SSR because of its large number, but comparing thousands of SNPs with just 8 SSR to make this point is neither fair nor necessary.**

**RESPONSE:** We definitely agree that 8 microsatellites are not comparable to the power of a large set of SNPs. However the point we intend to make in this study is valid in light of what has been published in the literature so far regarding the population genetics structure and diversity of A. angustifolia. Essentially all studies to date have used small sets of 5 to 10 microsatellites and this is the reason we made this comparison. This is in fact a crucial point of our MS, besides the SNP array development. It is therefore necessary, in our understanding, to clearly compare results obtained with the new array we present with was has been done so far to make the point that several of the conclusions provided in the literature for the population genetics of A. angustifolia are, in fact, misrepresentative.

To this end we made sure we genotyped the same exact individuals with the two methods so that no doubt would persist to the reader regarding population and individual sampling and the conclusions derived thereof regarding diversity, inbreeding and structure of the species. So we thank the reviewer for the comment but we do think it is necessary to keep this analysis exactly to make a strong point about this issue. We go over this in detail in our discussion especially in the final section “SNP array data to advance Araucaria genetics, conservation and breeding”. We discuss that the consequences of deriving conclusions about diversity and structure from microsatellite data could be deceptive to any recommendation for conservation.

**REVIEWER #2 COMMENT 5. line 486, “Northern populations were on average 25% less genetically diverse (Ho = 0.262) than southern populations (Ho= 0.353) based on SNPs, but this pattern was not as clearly detected with the microsatellite dataset” , I am not sure if this conclusion is correct. Based on statistical tests, I guess they all have significant differences.**

**RESPONSE:** Thanks for the comment and observation. We agree that the difference also existed but was quite smaller. We improved this sentence in the revised version by stating: *“Northern populations were on average 25% less genetically diverse (Ho = 0.262) than southern populations (Ho= 0.353) based on SNPs, but this pattern was less pronounced with the microsatellite dataset for which the difference was only 15% (Northern Ho = 0.587; Southern Ho = 0.694) (Table 3).”*
